# Root-Derived Endophytic Diazotrophic Bacteria *Pantoea cypripedii* AF1 and *Kosakonia arachidis* EF1 Promote Nitrogen Assimilation and Growth in Sugarcane

**DOI:** 10.3389/fmicb.2021.774707

**Published:** 2021-12-15

**Authors:** Rajesh Kumar Singh, Pratiksha Singh, Dao-Jun Guo, Anjney Sharma, Dong-Ping Li, Xiang Li, Krishan K. Verma, Mukesh Kumar Malviya, Xiu-Peng Song, Prakash Lakshmanan, Li-Tao Yang, Yang-Rui Li

**Affiliations:** ^1^Key Laboratory of Sugarcane Biotechnology and Genetic Improvement (Guangxi), Ministry of Agriculture, Sugarcane Research Center, Chinese Academy of Agricultural Sciences, Guangxi Key Laboratory of Sugarcane Genetic Improvement, Sugarcane Research Institute, Guangxi Academy of Agricultural Sciences, Nanning, China; ^2^Guangxi Key Laboratory of Crop Genetic Improvement and Biotechnology, Nanning, China; ^3^School of Marine Sciences and Biotechnology, Guangxi University for Nationalities, Nanning, China; ^4^State Key Laboratory of Conservation and Utilization of Subtropical Agro-Bio Resources, College of Agriculture, Guangxi University, Nanning, China; ^5^Microbiology Institute, Guangxi Academy of Agricultural Sciences, Nanning, China; ^6^Interdisciplinary Center for Agriculture Green Development in Yangtze River Basin, College of Resources and Environment, Southwest University, Chongqing, China; ^7^Queensland Alliance for Agriculture and Food Innovation, The University of Queensland, St Lucia, QLD, Australia

**Keywords:** antifungal activity, endophytes, nitrogen fixation, *Pantoea cypripedii*, *Kosakonia arachidis*, PGP, colonization, sugarcane

## Abstract

Excessive, long-term application of chemical fertilizers in sugarcane crops disrupts soil microbial flora and causes environmental pollution and yield decline. The role of endophytic bacteria in improving crop production is now well-documented. In this study, we have isolated and identified several endophytic bacterial strains from the root tissues of five sugarcane species. Among them, eleven Gram-negative isolates were selected and screened for plant growth-promoting characteristics, i.e., production of siderophores, indole-3-acetic acid (IAA), ammonia, hydrogen cyanide (HCN), and hydrolytic enzymes, phosphorus solubilization, antifungal activity against plant pathogens, nitrogen-fixation, 1-aminocyclopropane-1-carboxylic acid deaminase activity, and improving tolerance to different abiotic stresses. These isolates had *nifH* (11 isolates), *acdS* (8 isolates), and *HCN* (11 isolates) genes involved in N-fixation, stress tolerance, and pathogen biocontrol, respectively. Two isolates *Pantoea cypripedii* AF1and *Kosakonia arachidis* EF1 were the most potent strains and they colonized and grew in sugarcane plants. Both strains readily colonized the leading Chinese sugarcane variety GT42 and significantly increased the activity of nitrogen assimilation enzymes (glutamine synthetase, NADH glutamate dehydrogenase, and nitrate reductase), chitinase, and endo-glucanase and the content of phytohormones gibberellic acid, indole-3-acetic acid, and abscisic acid. The gene expression analysis of GT42 inoculated with isolates of *P. cypripedii* AF1 or *K. arachidis* EF1 showed increased activity of *nifH* and nitrogen assimilation genes. Also, the inoculated diazotrophs significantly increased plant nitrogen content, which was corroborated by the ^15^N isotope dilution analysis. Collectively, these findings suggest that *P. cypripedii* and *K. arachidis* are beneficial endophytes that could be used as a biofertilizer to improve plant nitrogen nutrition and growth of sugarcane. To the best of our knowledge, this is the first report of sugarcane growth enhancement and nitrogen fixation by Gram-negative sugarcane root-associated endophytic bacteria *P. cypripedii* and *K. arachidis*. These strains have the potential to be utilized as sugarcane biofertilizers, thus reducing nitrogen fertilizer use and improving disease management.

## Introduction

Global food security is a major sustainable development goal of United Nations. This is also a major challenge for developing countries with limited resources and scientific capacity. Agriculture is a major consumer of energy and cause of environmental damages, mainly due to the large input of fertilizers, land use practices, and use of fossil fuel (Fraser and Campbell, 2019; Yang et al., 2021). Sustainable intensification of agriculture is a well-recognized concept and is being practiced in the developed world (Tilman et al., 2002; Vanlauwe et al., 2019). It improves resource use efficiency, reduces agricultural inputs, especially fertilizers and other agri-chemicals, and expansion of mechanized farm operations. In this context, considerable research to understand and exploit soil and rhizosphere microbiomes and plant endophytes to reduce fertilizer input and suppress pathogens are now underway globally. This is particularly relevant when 60–90% of the applied chemical fertilizers are wasted depending on the crop and region, and the manufacturing of agri-chemicals like nitrogen (N) fertilizers is a highly energy-intensive process (Bhardwaj et al., 2014). Biofertilizers, which are live cells of microbes, are a potential alternative to chemical fertilizers since they provide nutrients to plants, reduce soil-borne diseases, and improve the health and quality of soil (Bhardwaj et al., 2014; Kour et al., 2020).

Sugarcane (*Saccharum* spp. interspecific hybrids), a member of Poaceae, is a major commercial crop grown in the tropical and subtropical areas of the world. Due to its large contribution to sugar production, manufacture of ethanol, and a source of environmentally sustainable green energy, it is an economically important crop worldwide (Vital et al., 2017). It accounts for more than 80% of global sugar production, with Brazil, India, China, and Thailand accounting for 60% of total output (FAO, 2020a). Global sugar output was around 166.18 million metric tons during the 2019–2020 (United States Department of Agriculture [USDA], 2020). China is the world’s third largest producer of sugar, with an annual output of over 13 million tons. Sugarcane accounts for more than 90% of sugar production in China, with Guangxi province accounting for over 65% of total production (Li et al., 2016). Chinese sugarcane crop productivity, however, is lower than the world average. This is largely caused by sub-optimal crop production management and widespread occurrence of diseases and pests (Li et al., 2016). Endophytic bacteria, which survive and grow inside plant tissue, have been extensively studied for control of diseases and amelioration of stresses in a variety of plants (Kumar et al., 2017). The use of plant growth-promoting endophytic bacteria (PGPEB), can therefore be an effective tool for improving crop growth and productivity under non-stresses as well as challenging environmental conditions, including poor soil fertility.

Nitrogen fixation is an important source of N for many crops and both plant growth-promoting bacteria (PGPB) growing on the root surface and PGPEB facilitate plant N availability (Sur et al., 2010; Leghari et al., 2016; Oleńska et al., 2020). The crop productivity depends on N, and use of inorganic N for crop production is still increasing at a global scale (FAO, 2020b) and it undermines efforts to mitigate climate change. The utilization of biological N fixation (BNF) to minimize the input of N fertilizers in sugarcane has been reported, though the BNF microbes are not well characterized. Different bacterial genera such as *Azotobacter*, *Azospirillum, Bacillus*, *Pseudomonas*, and *Enterobacter*, have been shown to be associated with BNF (Martins et al., 2020). The use of PGPEB to improve crop production may have advantages over epiphytic and rhizosphere bacteria as they are more vulnerable to soil and other external conditions (James, 2000). These bacteria produce several phytohormones (auxin, cytokinin, ethylene, and gibberellins) and growth-enhancing compounds (siderophore, hydrogen cyanide, fixed N, and hydrolyzing enzymes) and can be used as biofertilizers (Stefan et al., 2013; Mihalache et al., 2015; Li et al., 2017; Raklami et al., 2019). Our previous research showed BNF in certain varieties of commercially grown sugarcane, and we have identified some rhizobacteria involved in BNF from those varieties (Li et al., 2017; Singh et al., 2020b,c). This research is now extended to study the endophytic bacteria from sugarcane roots, to assess their plant growth-promoting (PGP) potential, including their ability for BNF.

Here, we report the isolation and characterization of non-pathogenic endophytic *Pantoea* and *Kosakonia* diazotrophic bacteria colonized in sugarcane roots. The genera *Pantoea* and *Kosakonia*, belong to Enterobacteriaceae family, are non-spore-forming, rod-shaped, and Gram-negative bacteria (Tambong, 2019). Members of *Pantoea* and *Kosakonia* genera are known to interact and elicit beneficial effects on plant growth (Brock et al., 2018; Chen and Liu, 2019; Romano et al., 2020; Singh et al., 2020b, 2021b; Suman et al., 2020), but little is known about their potential for disease control and N-fixation in sugarcane. Considering the widespread occurrence of diseases and the large reduction in N input required in sugarcane crops in China, we studied the disease control and BNF properties of members of *Pantoea* and *Kosakonia* genera, and the results are presented here.

## Materials and Methods

### Sampling and Endophytic Bacteria Isolation

Five sugarcane species, *Saccharum officinarum* L. cv Badila, *Saccharum barberi* Jesw. cv Pansahi, *Saccharum robustum*, *Saccharum spontaneum*, and *Saccharum sinense* Roxb. cv Uba were used for this study. All plants were obtained from the experimental farm of Sugarcane Research Institute, Guangxi Academy of Agricultural Sciences, Nanning, Guangxi (latitude 22° 50′ N, longitude 108° 14′ E, and elevation 70 m), China. The climate in the study region was humid subtropical, with an annual mean temperature of 21.83°C and 1,290 mm of rainfall. At the plant elongation stage, root samples were taken for the isolation of endophytic bacteria according to Dobereiner et al. (1993) method. The soil adhering to the roots was cleaned by thoroughly washing them with tap water, and then rinsed with sterile distilled water, 75% ethanol (5 min), and sodium hypochlorite solution (3% for 5 min). Five root samples were chosen from each sugarcane species. The root samples with white tips, which indicated continued growth, were used to isolate bacteria. Root pieces (1 gm per clone) were transferred to cold, sterilized mortal and pestles, and crushed with 1 mL of sterile 5% sucrose solution. An aliquot of 100 μL from each sample was spread on the different medium (Supplementary Table 1) and incubated for 3–5 days at 30 ± 2°C. After incubation, morphologically different bacterial colonies were chosen. The isolated strains were kept at −20°C in a 25% glycerol solution.

### Identification of Endophytic Isolates and Analysis of *nifH, acdS*, and Hydrogen Cyanide Biosynthetic Genes

The strains were identified by analyzing their 16S *rRNA* gene sequences amplified from genomic DNA using universal primers pA and pH (Supplementary Table 2) (Edwards et al., 1989), and conditions as described by Singh et al. (2021b). Briefly, pure bacterial cultures were grown in Luria-Bertani (LB) broth for 48 h on a shaker incubator and maintained at 32 ± 2°C for 160 rpm. Genomic DNA was isolated from 1.5 mL broth culture using a DNA extraction kit (CWBIO, Beijing-China) following the manufacturer’s instructions. The PCR amplification of the 16S *rRNA* gene was completed and the amplified PCR products were purified by using a BioFlux kit (Hangzhou, China) and then sequenced by Sangon Biotech (Shanghai, China). Phylogenetic analysis was performed to verify identities and determine the evolutionary relationship of the isolates with reference strains from the GenBank public database and aligned by ClustalW. Phylogenetic trees were created by MEGAX for the 16S *rRNA* gene (Kumar et al., 2018) via Neighbor-Joining method (Saitou and Nei, 1987). The evolutionary distances were calculated by using the neighbor-joining method (Nei and Kumar, 2000). The bootstrap study (1,000 replicates) was carried out as described earlier (Felsenstein, 1985).

The primer sequences shown in Supplementary Table 2 were used for the amplification of *nifH* (Poly et al., 2001), *acdS* (Li et al., 2011), and HCN (Ramette et al., 2003) for all selected strains.

### Acetylene Reduction Assay and 1-Aminocyclopropane-1-Carboxylate Deaminase Activity

*In vivo* nitrogenase activity of all bacterial isolates was determined by acetylene reduction assay (ARA) by inoculating pure culture in 10 mL of McCartney vial comprising semi-solid N-free medium and incubated at 30 ± 2°C for 48 h (Hardy et al., 1968).

1-aminocyclopropane-1-carboxylate deaminase (ACCD) activity of bacterial isolates was measured based on their capability to use ACC (3 mM) as a sole N source in the Dworkin and Foster (DF) salt minimal medium (Penrose and Glick, 2003). All strains were spot inoculated on (i) Petri plates comprising DF salts minimal medium containing ACC, (ii) DF minimal medium without of ACC (negative control), and (iii) DF minimal medium with (NH_4_)_2_SO_4_ (2 g L^–1^) (positive control). And, growth was compared with controls after incubation at 32 ± 2°C for 5–7 days. Strains that showed good growth on ACC plates were chosen for quantitative analysis, following Honma and Shimomura (1978) protocol.

### Qualitative and Quantitative Evaluation of Plant Growth-Promoting Characteristics of Bacterial Isolates

#### Phosphate Solubilization

An aliquot of ∼10 μL of freshly produced bacteria was spotted on Pikovskayas agar medium to study the phosphate (P) solubilization capability (Hi-Media). The spotted plates were placed at 30 ± 2°C for 3–5 days and detected for the development of a clear zone around the bacterial colony.

For quantitative P-solubilization, each bacterial isolate was inoculated in 100 mL Erlenmeyer flasks containing 25 mL of Pikovskaya’s medium (≈10^8^ CFU mL^–1^) and incubated in a shaker (180 rpm) at 30 ± 2°C for 72 h. Autoclaved medium (uninoculated) served as control. 20 mL of each culture was collected after 72 h of growth and centrifuged for 10 min at 13,000 *g* to obtain cell-free supernatants. The amount of phosphorus in the culture supernatant was measured using Fiske and Subbarow (1925). The pH of the bacterial broth was also measured with a digital pH meter.

#### Hydrogen Cyanide Production

The Hydrogen cyanide (HCN) produced by each endophytic bacterial isolate was determined by Lorck (1948) process. Briefly, pure bacterial isolate in LB medium with 4.4 g glycine L^–1^ was inoculated in 15 mL broth in a test tube. A sterile Whatman filter paper no. 1 soaked in picric acid (1%) solution was hung in the test tubes once dried. The test tubes were then closed with parafilm and kept for 5–10 days at 30 ± 2°C. After incubation, the change in the color of the filter paper, i.e., yellow to orange-brown, or reddish-brown shows the production of cyanide from bacterial strains.

#### Siderophore Production

To measure the siderophore production by bacterial isolates, both qualitative and quantitative methods were used. The capacity of bacterial isolates to produce siderophores was evaluated using the universal Chrome azurol S (CAS) agar medium (Schwyn and Neilands, 1987). The pure freshly produced all bacterial isolates were dotted on Petri plates with CAS medium and cultured for 4–5 days at 30 ± 2°C. The formation of an orange zone (hydroxamate-type siderophore) or a purple zone (catechol-type siderophore) surrounding the bacterial colonies on the plates was used to determine siderophore production by endophytic bacteria.

Quantitative estimation (hydroxamate-type siderophore) was completed by taking the supernatant of bacterial cultures grown in a broth of LB medium (Hu and Xu, 2011). Screwcap tubes (20 mL) containing 5 mL of LB broth were autoclaved. Afterward, 10 μL of freshly grown bacterial suspension (≈10^8^ CFU mL^–1^) was inoculated, and the uninoculated broth was maintained as the control. After incubation at 30 ± 2°C for 3 days, bacterial cultures were pelleted by centrifuging at 12,000 rpm for 10 min. at 5°C, and the clear supernatant was used to determine the production of siderophore. 0.5 mL supernatant of each bacterial isolate was mixed with 0.5 mL CAS solution, kept in dark condition for 20 min, and measured its optical density at 630 nm by SPARK^®^ multimode microplate reader (Model- SW Sparkctl. Magellan V2.2 STD 2PC, Austria). Payne (1993) method was used to determine siderophore production in percent siderophore unit (PSU).

#### Test for Colorimetric Indole-3-Acetic Acid (IAA) Detection

Indole-3-acetic acid synthesis capacity of all isolates were measured the colorimetric method of IAA quantitation as described by Gordon and Weber (1951). Overnight bacterial strains were cultured in LB broth medium at 32 ± 2°C with shaking at 180 rpm after inoculum was prepared (≈10^8^ CFU mL^–1^). The inoculum was added in LB broth medium supplemented with L-tryptophan (0.5 and 1.0 g L^–1^) as the IAA precursor and incubated for 7 days at 30 ± 2°C. Subsequently, bacterial cells were separated by centrifugation, and the supernatant was used for quantitative measurement of IAA-producing bacteria by using Salkowski’s reagent.

#### Ammonia Production

Freshly cultured bacterial strains were grown in peptone water broth for 5 days at 30 ± 2°C and supernatant was used to measure ammonia production using Nessler’s reagent following the method described previously (Goswami et al., 2014). The concentration of ammonia was calculated using the ammonium sulfate standard curve, which ranged from 0.1 to 1 μ moL mL^–1^.

### Biocontrol Assay of Endophytic Bacteria

Using the dual-culture method established by Singh et al. (2014), the chosen bacterial isolates were evaluated for *in vitro* antifungal activity against *Fusarium verticillioides* (*FV*), and *Fusarium oxysporum* f. sp. *cubense* (*FOC*). A fungal disk (5 mm) was placed in the center of a potato dextrose agar (PDA) and nutritional agar (NA) (1:1) plates and bacterial isolates suspended in LB broth (∼6 × 10^8^ cell mL^–1^) were spotted 3 cm away from the fungal disk. Plates with fungal disks (without bacterial isolates) were applied as a control. The plates were maintained at 28 ± 2°C for 5–7 days. By comparing the development of fungal mycelia with and without the tested bacterial isolates, the antifungal activity was determined.

To examine the cell-free crude extract of chosen (dual-culture method) bacteria against the pathogens, strains were cultured in *LB broth* medium for 5–7 days at 120 rpm and 32 ± 2°C on an orbital shaker incubator. The bacterial cells were separated from the broth medium by centrifugation (14,000 rpm for 15 min at 4°C) and filtered using a sterilized membrane (0.22 m pore size; Merck Millipore Ltd.), and the filtrate was stored at −20°C until further use. The pathogen spores were scraped and suspended in sterile distilled water (10 mL) and diluted the spore suspension (10^6^ CFU mL^–1^; colony-forming units), then distributed on Petri dishes comprising PDA. A 5-mm diameter hole was made into the medium with a cork borer, and the well was sealed with sterilized agarose (0.2%). Once the agarose was set and dried, 100 μL of cell-free culture filtrate and LB *broth* medium (control) was applied. The plates were incubated at 26 ± 2°C for 3–5 days.

### Bacterial Production of Cell Wall Degrading Enzymes

Use of hydrolytic enzymes is a biocontrol strategy utilized by many microorganisms to restrict the development of fungal pathogens. In this study, the production of hydrolytic enzymes such as chitinase (MM1062O1), protease (MM1206O1), β- 1,3 glucanase (MM91504O1), and cellulase (MM91502O1) by selected strains was studied (Guo et al., 2020). A single strain of freshly grown bacteria was inoculated into 20 mL of LB medium and maintained at 32 ± 2°C for 36–48 h in an incubator shaker, after which the supernatant was centrifuged at 12,000 rpm for 10 min at 4°C and utilized for different enzyme activity measurement using enzyme-linked immunosorbent assays (ELISA) (Wuhan Colorful Gene Biological Technology Co. Ltd., China).

### Scanning Electron Microscopy

Biocontrol interaction studied of the fungal plant pathogens and selected diazotrophs were done by scanning electron microscopy (SEM). A small piece of hyphae (∼3 mm) was cut at an interaction point into the plate and control plate only fungal pathogen, fixed in 2% glutaraldehyde for 4 h at room temperature, then rinsed three times with phosphate buffer (0.1 M, pH 7.4) for 15 min and after transferred into blocks with 1% OsO_4_ in 0.1 M PB (pH 7.4) for 1–2 h at 20°C. Then, blocks were washed with 0.1 M phosphate buffer (0.1 M, pH 7.4) for 15 min. The samples were desiccated with different concentrations of ethanol, i.e., 30, 50, 70, 80, 90, 95, and 100% for 15 min and at last with isoamyl acetate for 15 min. Samples were dried in a critical point dryer (model K850 Quorum) by a carbon sticker sputter-coated with gold-palladium for the 30 s. Samples were observed with SEM (HITACHI, SU8100, Japan). This technique is also used to study the colony morphology of selected diazotrophs as well as to verify the colonization in different tissues (root and leaf) of sugarcane plants.

### Stress Resistance Test of Endophytic Bacteria

*In vitro* screening of endophytic isolates for abiotic stress tolerance was analyzed with a broad range of temperatures (20–45°C), pH (5–10), and different NaCl concentrations (7–12%) (Sharma et al., 2019, 2021). 100 μL (≈10^8^ CFU mL^–1^) of fresh cultures were transferred in 5 mL LB broth medium and incubated in a gyratory shaker set at 120 rpm for 36 h at 32 ± 2°C, and growth was measured at 600 nm using SPARK^®^ multimode microplate reader (Model- SW Sparkctl. Magellan V2.2 STD 2PC, Austria).

### Colonization Pattern of Selected Diazotrophs in the Sugarcane Plant

#### Plasmid Transformation

For this experiment, we selected strains AF1 and EF1, the most potential isolates based on the above studies. Both isolates were resistant to ampicillin and taken as recipients with GFP-pPROBE-pTetr-OT tagging sensitive to kanamycin. Plasmid pPROBE-pTetr-OT comprising the green fluorescent protein (GFP) gene expressed under the Tet*^r^* promoter was inserted by biparental mating with donor strain *Escherichia coli* TG1 (Lin et al., 2012). The plasmid has a wide host range and could be quantified in both Gram-positive and Gram-negative bacteria. In an incubator shaker, the recipient and donor isolates were combined in a 1:2 ratio and maintained at 30 ± 2°C for 48–72 h. A 100 μL aliquot of the above mixture was spread onto LB agar plate and kept overnight at 30 ± 2°C and strains displaying green fluorescence under UV illumination were used for further study.

#### Inoculation of Micro-Propagated Sugarcane Plantlets

Micro-propagated sugarcane plantlets (variety GT42) were procured from Sugarcane Research Institute, Guangxi Academy of Agriculture Sciences (GXAAS), Nanning, China. Five separate plantlets were moved into a glass bottle containing 50–75 mL of MS liquid medium with sucrose and basal salt mixture. After, 3 days sugarcane plantlets were shifted in other autoclave bottles comprising GFP-tagged bacterial suspension (2.0 × 10^6^ mL^–1^), and without suspension have been prepared for utilizing as a control. All plantlets were grown in a growth chamber at 30°C with a 14 h photoperiod at 60 μmoL m^–2^ s^–1^ photon flux density.

#### Laser Scanning Confocal Microscopy

Afterward, 4–5 days sugarcane plantlets both inoculated and un-inoculated were carefully removed from the bottles and washed with autoclaved distilled water, then dried at room temperature. Sugarcane plantlets tissues (root, leaf, and stem) were cut into small parts and mounted on a clean glass slide under a coverslip. Different tissues of sugarcane plantlets were viewed with a Leica DMI 6000 microscope (Mannheim, Germany) using a confocal scanning laser microscope (Olympus SXZ16) at different emission lengths.

### Plant Inoculation Studies of Potential Diazotrophic Isolates, i.e., a Pot Experiment

#### Experimental Design and Treatments

The pot experiment was performed in January, 2020, at the Sugarcane Research Institute, GXAAS, Nanning. Sugarcane variety GT42 was used to establish the interactions between diazotrophic isolates and the host plant. The trial was completed in a greenhouse and the seed canes of test variety were obtained from Sugarcane Research Institute. A disease-free seed cane was used for this experiment. Hot-water treatments of sugarcane stalks (45–50°C for 2 h) were used to disinfect seed cane. Soil was collected from the top 2–20 cm soil depth from the experimental field sites of Sugarcane Research Institute and air-dried, crushed, and its physicochemical properties were analyzed. A plastic pot (30 cm diameter and 40 cm deep) holding 20 kg of soil and sand mixture (3:1 w/w) were used. Approximately similar size and shape of sugarcane plantlets at a three-leaf stage were carefully removed from nursery plants. And finally, the roots were washed slowly with flowing tap water then put in a tub and rinsed carefully until the root was cleaned. The experiment was accomplished in a complete randomized block design with five biological repeats and each pot contained two treated plantlets. The three treatments comprised of: (1) without inoculation-control (WI), (2) inoculation with *P. cypripedii* (AF1), and (3) Inoculation with *K. arachidis* (EF1). The diazotrophic bacteria were cultured in LB medium for 24–36 h (200 rpm, 32 ± 2°C) and centrifuged. Pellet was dissolved with autoclaved distilled water and prepared a bacterial cell suspension were adjusted at 10^8^ CFU mL^–1^, and mixed with 1% autoclaved carboxymethyl cellulose (CMC) solution. All plantlets of sugarcane roots were immersed in CMC solution for 1 h. Following these treatments, plantlets were plotted into plastic pots and kept under controlled environmental conditions at 14 h day/10 h night; 60–70% relative humidity, and 26°C/20°C day/night temperature.

#### Plant’s Harvest, RNA Extraction, Purification, and cDNA Synthesis

Isolation of total RNA of root (100 mg) was done with Trizol reagent (Tiangen, China), and purified by RNeasy Plant Mini Kit (Qiagen), following the manufacturer’s commands. Extracted RNA samples were processed with DNase I (Promega, United States) to eliminate contaminating DNA and quantified using a Nano photometer (Pearl, Implen-3780, United States). The Prime-Script™ RT Reagent Kit (TaKaRa, Dalian, China) was used to synthesize single-stranded cDNA from 1 g of total RNA, consistent with the manufacturer’s guidelines.

#### cDNA Amplification

Amplification of the cDNA sequences employed the expression of aminomethyl transferase*- AMT*, nitrate transporter*- NRT*, Nitrate reductases- *NR*, Glutamate synthase- *GS*, Glutamine synthetase- *GOGAT*, N fixation-*nifH*, endo-glucanase- β*-1, 4-GA*, and chitinase- *CHI*, genes in the root tissues of sugarcane during plant-microbes interaction at tillering phase were analyzed in greenhouse condition after treatment with strains (AF1 and EF1) in GT42 sugarcane varieties with control plants. The primer sequences used in this study are presented in Supplementary Table 2. The qRT-PCR reaction mixture consisted of SYBR Premix Ex Tap™ II (TaKaRa, Japan), 10 μL of SYBR Premix, 1 μL of each primer (10 μM), 2 μL of RNA template (10 × diluted cDNA), and 6 μL of ddH_2_O in a total volume of 20 μL with five repeats in Real-Time PCR Detection System (Bio-Rad, United States). Amplification was started with a denaturation step of 94°C for 2 min, followed by 40 cycles of 94°C for 30 s, 60°C for 20 s, and 72°C for 30 s, and the last extension at 72°C for 2 min. To standardize qRT-PCR data, the housekeeping gene glyceraldehyde 3-phosphate dehydrogenase (GAPDH) was utilized as the reference gene, and the relative expression of all genes was quantified using the 2^–ΔΔCt^ technique (Livak and Schmittgen, 2001).

#### ^15^N Abundance Plant Analysis

The ^15^N isotope dilution method quantifies the biological N fixation in the GT42 sugarcane variety (Singh et al., 2020a). This method involves the testing of N-fixing crops with the inoculation of AF1 and EF1 isolates. Soil and sand were mixed with a ratio of 1:3 (w/w) and sterilized two times for 45 min at 121°C. After cooled at room temperature 10 mg ammonium sulfate-^15^N (10.12 percent atom ^15^N excess) per kg of soil was added and homogenized for proper distribution of ^15^N. Twenty kg of ^15^N soil-sand mixture was filled in the pots and each pot contains two treated sugarcane plantlets for GT42. This experiment was performed in a completely randomized block design with five biological repetitions, and comprised three treatments: (1) without bacterial inoculation (control) (2) inoculation with *P. cypripedii* (AF1), and (3) inoculation with *K. arachidis* (EF1) of each variety. At the tillering phase, plants were harvested and washed with distilled water to eliminate the soil attached to the roots and plants. Roots, leaves, and stems were separated and ground to a fine powder. Five milligrams of powdered root, leaf, and stem materials for all samples were analyzed for ^15^N isotope content using K05 automatic Kjeldahl N determination equipment (Shanghai Sonnen automated research instrument co. Ltd.), and elementary analysis was done by isotope ratio mass spectrometers (Thermo Fisher Delta V Advantage IRMS). The contribution of N derived from the air (Ndfa) in different tissues of all sugarcane varieties was calculated by Urquiaga et al. (1992).

#### Determination of Physiological Parameters and Enzymes Associated With Nitrogen Metabolism, and Biocontrol, and Phytohormone Analysis

The experimental plants were collected at the tillering stage. The growth parameters of sugarcane plants including plant height, fresh weight (root and shoot), leaf area (Cl 203 Handheld laser leaf area meter, Bio-Science), chlorophyll content (Chlorophyll meter; SPAD-502 Plus; Konica Minolta Inc.; Japan), net photosynthetic rate, transpiration rate, and stomatal conductance (LI-6,800 compact portable photosynthesis system) were recorded.

Also, the activity of different N-metabolism enzymes (glutamine synthetase-GS, NADH glutamate dehydrogenase, and nitrate reductase-NR), biocontrol-related enzymes (β-1,4 and β-1,3 glucanase-GLU, and chitinase-CHI) (Singh et al., 2021c), and hormones (Gibberellins- GA_3_, Indole-3-acetic acid- IAA, and Abscisic acid- ABA) were extracted (Singh et al., 2018) and analyzed by plant ELISA kit (Colorful Gene Biological Technology Co. Ltd., Wuhan, China) according to the manufacturer’s guidelines.

### Statistical Analysis

All tests were carried out in three repetitions. The standard error was computed using mean values, and the statistical significance threshold was set at *p* ≤ 0.05. MS Excel 2016 was utilized for basic statistical analysis of data, i.e., means standard deviation, and bar graphs. Analysis of variance was used to evaluate the statistical significance of the experimental data, followed by multiple comparisons using Tukey’s HSD test. A heatmap was also prepared following the method described by to Babicki et al. (2016), a heat map was created.

## Results

### Isolation of Endophytic Bacteria

A total of 175 endophytic bacterial strains were isolated from the roots of five sugarcane species Among these, only 11 Gram-negative strains of two genera *Pantoea* and *Kosakonia* were selected based on partial sequencing of the 16S *rRNA* gene. Further, the increase in interest in both genera for their potential PGP and nitrogenase activities was also prompted us to select these strains for the study.

### Molecular Identification of Endophytic Diazotrophic Bacteria

#### Sequencing of 16S rRNA Gene

In this present study, molecular identification of all diazotrophic isolates was accomplished through the amplification of 16S *rRNA* gene sequencing (∼1.4–1.5 kb) with the primer’s pA and pH. Using BLAST-N searches with type strain database, we found that all the strains belong to the genus *Pantoea* and *Kosakonia* with *rRNA* sequences similarity values at ≈97.77–99.58%. All sequences of the isolates were deposited to NCBI GenBank under MZ497007 - MZ497017 accessions numbers (Table 1).

**TABLE 1:** Identification of selected isolates through 16S *rRNA* gene and their nitrogenase activity via acetylene reduction assay, hydrogen cyanide production, and the presence of gene amplification with an accession number.

Isolates	Strain name	16S accession numbers	ARA (nmoL C_2_H_4_ mg protein h^–1^)	*nifH* gene	*nifH* accession numbers	HCN production	HCN gene	HCN accession numbers
AE2	*Pantoea dispersa*	MZ497007	15.30 ± 0.23^e^	+	MZ502257	+	+	MZ502268
AE3	*Kosakonia oryzae*	MZ497008	10.29 ± 0.15^g^	+	MZ502258	−	+	MZ502269
AF1	*Pantoea cypripedii*	MZ497009	23.24 ± 0.35^b^	+	MZ502259	+	+	MZ502270
BA4	*Pantoea ananatis*	MZ497010	18.64 ± 0.28^c^	+	MZ502260	+	+	MZ502271
BB2	*Pantoea allii*	MZ497011	12.59 ± 0.19^f^	+	MZ502261	−	+	MZ502272
CF1	*Pantoea agglomerans*	MZ497012	10.08 ± 0.15^g^	+	MZ502262	−	+	MZ502273
EA1	*Kosakonia radicincitans*	MZ497013	10.50 ± 0.16^g^	+	MZ502263	+	+	MZ502274
EF1	*Kosakonia arachidis*	MZ497014	35.14 ± 0.52^a^	+	MZ502264	+	+	MZ502275
ACCR4	*Kosakonia oryziphila*	MZ497015	9.46 ± 0.14^h^	+	MZ502265	+	+	MZ502276
ACCR21	*Kosakonia quasisacchari*	MZ497016	17.39 ± 0.26^d^	+	MZ502266	+	+	MZ502277
ACCE1	*Kosakonia pseudosacchari*	MZ497017	6.95 ± 0.10^i^	+	MZ502267	−	+	MZ502278

*Means followed by the similar letter are not significantly different (p ≤ 0.05).*

### Phylogenetic Analysis

**FIGURE 1:**
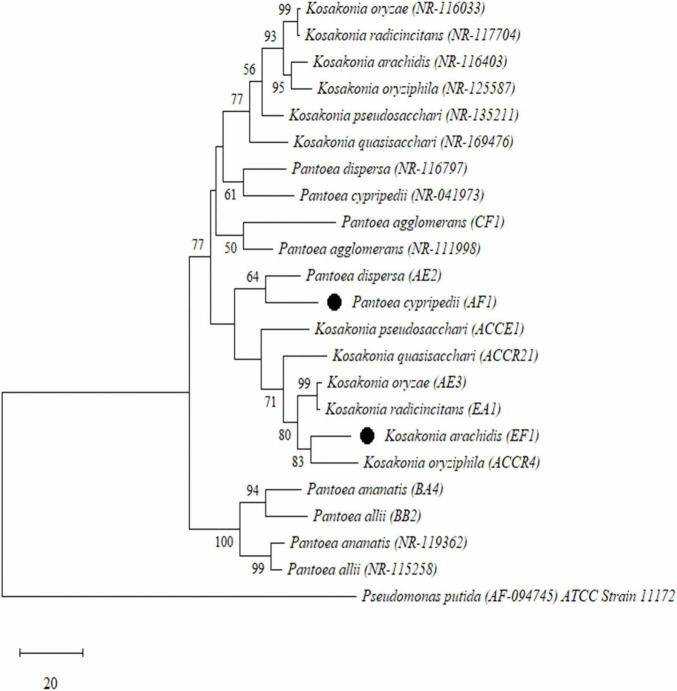
Phylogenetic tree analysis based on the partial 16S rRNA gene sequences of all selected Gram-negative endophytic nitrogen-fixing bacterial isolates isolated from sugarcane root. Scale bar means the number of changes per base location and *Pseudomonas putida* was used as an outgroup.

The sequences of the 16S *rRNA* gene were aligned and used to build a phylogenetic tree using the neighbor-joining method. We found that all isolates were distributed into four major clusters using a total of 1,000 bootstrap samples of representative isolates compared to type strains of related taxa. According to this, all endophytic diazotroph bacterial isolates were grouped into different clusters with cluster I formed with two isolates (BA4- *Pantoea ananatis* and BB2- *Pantoea allii*), cluster II also formed with two isolates (AE2- *Pantoea dispersa* and AF1- *P. cypripedii*), cluster III formed with one isolate (CF1- *Pantoea agglomerans*), and cluster IV formed with six isolates (AE3- *Kosakonia oryzae*, EA1- *Kosakonia radicincitans*, EF1- *K. arachidis*, ACCR4- *K. oryziphila*, ACCE1- *K. pseudosacchari*, and ACCR21- *Kosakonia quasisacchari*) as shown in Figure 1.

### Detection of *nifH*, *acdS*, and *HCN* Genes

The PCR-based molecular detection of *nifH*, *acdS*, and *HCN* genes that promote plant development directly or indirectly was studied. Results showed that all strains had positive *nifH* gene amplification (Supplementary Figure 1A), and the evolutionary relationship of the *nifH* phylogenetic tree is presented (Supplementary Figure 1D). After sequencing, all sequences were matched with *nifH* gene sequences obtained from NCBI by BLASTN search. The *nifH* sequences of test isolates were submitted to NCBI GenBank with accession numbers MZ502257 – MZ502267 (Table 1).

Amplification of the *acdS* gene revealed that nine strains had ACC deaminase gene (Supplementary Figure 1B). The HCN gene was present in all 11 strains at ∼587 bp of amplification (Supplementary Figure 1C). All sequences showed 95–100% similarity with other HCN genes present in the NCBI database. Accession numbers of HCN genes of seven isolates are MZ502268 – MZ502278 (Table 1).

### Nitrogenase Activity

The nitrogenase activity obtained from isolates varied from 6.95 ± 0.10 to 35.14 ± 0.52 C_2_H_4_ mg protein h^–1^ nmoL (Table 1). The maximum nitrogenase activity was obtained in EF1 followed by AF1, BA4, ACCR21, and AE2 (which is ≥15 nmoL C_2_H_4_ mg protein h^–1^, respectively). Isolate ACCE1 exhibited ≤10 nmol C_2_H_4_ mg protein h^–1^ level of nitrogenase activity as compared with other isolates (Table 1).

### 1-Aminocyclopropane-1-Carboxylate Deaminase Activity

1-aminocyclopropane-1-carboxylate deaminase activity was measured to determine isolates’ ability to utilize ACC as a nitrogen source to grow. After 4–5 days of incubation at 32 ± 2°C, all of the endophytic isolates were able to grow on medium supplemented with 3 mMoL L^–1^ of ACC (Table 2). Based on this result, the ACCD enzyme activity was determined quantitatively and the amount of α-ketobutyrate breakdown during ACC by the ACCD enzyme. It was observed that AF1, EF1, and AE2 isolates used maximum ACC and produced 1325.62 ± 19.67, 824.33 ± 12.23, and 676.70 ± 10.04 α-ketobutyrate μmoL mg^–1^ h^–1^, respectively (Table 2).

**TABLE 2:** *In vitro* screening for assessing the endophytic plant growth-promoting, 1-Aminocyclopropane-1-carboxylate deaminase activity and antifungal activities for selected bacterial isolates obtained from sugarcane root.

Isolates	ACC deaminase activity		Phosphate		Siderophore		Ammonia		Antifungal activities			
	A	B	A	B (PSI)	A	B (PSU)	A	B (μ moL mL^–1^)	C (FV)	D (*FV*)	C (*FOC*)	(D) (*FOC*)
AE2	+	676.70 ± 10.04^c^	−	−	+++	59.35 ± 1.19^b^	+++	6.18 ± 0.14^a^	56.67 ± 0.65^b^	5.8	64.71 ± 0.53^c^	6.1
AE3	+	221.35 ± 3.28^f^	++	2.81	++	39.16 ± 0.75^cd^	++	3.65 ± 0.10^f^	30.00 ± 1.04^e^	−	40.00 ± 0.89^h^	−
AF1	+	1325.62 ± 19.67^a^	++	2.68	+++	71.23 ± 1.66^a^	+++	6.33 ± 0.14^a^	64.44 ± 0.53^a^	5.1	74.12 ± 0.39^a^	5.5
BA4	+	359.11 ± 5.33^d^	−	−	++	32.60 ± 1.59^de^	+++	5.72 ± 0.13^b^	24.44 ± 1.12^f^	−	44.71 ± 0.82^g^	−
BB2	+	323.77 ± 4.80^e^	+++	3.34	+	06.57 ± 3.74^f^	+++	5.16 ± 0.12^cd^	20.00 ± 1.19^g^	−	29.41 ± 1.05^i^	−
CF1	+	209.68 ± 3.11^f^	++	2.54	++	40.37 ± 4.80^cd^	++	4.33 ± 0.11^e^	35.56 ± 0.96^d^	−	60.00 ± 0.60^d^	3.4
EA1	+	204.91 ± 3.04^f^	+	2.22	++	29.74 ± 1.22^e^	+++	4.74 ± 0.08^de^	44.44 ± 0.83^c^	−	55.29 ± 0.67^e^	−
EF1	+	824.33 ± 12.23^b^	++	2.77	+++	63.46 ± 1.16^ab^	+++	6.21 ± 0.13^a^	66.11 ± 0.50^a^	6.5	70.59 ± 0.44^b^	6.3
ACCR4	+	103.37 ± 1.53^g^	+	2.34	+	8.47 ± 3.33^f^	+	3.27 ± 0.09^f^	34.44 ± 0.98^d^	−	54.12 ± 0.68^e^	−
ACCR21	+	344.99 ± 5.12^de^	+++	3.56	++	23.90 ± 2.45^e^	+++	5.55 ± 0.12^bc^	45.56 ± 0.81^c^	−	52.94 ± 0.70^e^	−
ACCE1	+	93.35 ± 1.38^g^	−	−	++	43.89 ± 1.72^c^	+	2.37 ± 0.17^g^	23.33 ± 1.14^f^	−	49.41 ± 0.75^f^	−

*A, qualitative analysis; B, quantitative analysis (μmoL mg^–1^ h^–1^); (+++), high production; (++), medium production; (+), low production; PSI, phosphate solubilization index; PSU, percent siderophore unit; C, dual culture (% inhibition); D, well diffusion (mm), FV; Fusarium verticillioides, FOC; Fusarium oxysporum f. sp. cubense. Different letters indicate significant differences among treatments at p ≤ 0.05.*

### Plant Growth Promoting Traits of Endophytic Isolates

A total of 11 selected endophytic bacterial isolates (from AE2 to ACCE1) were tested for their potential PGP activities, such as the production of siderophores, phosphate, HCN, and ammonia (Tables 1, 2 and Supplementary Figure 2).

After 5–7 days of incubation, eight (73%) endophytic isolates displayed positive phosphate solubilization on the Pikovskaya agar medium plate containing Ca_3_(PO_4_)_2_, with a clear zone around the colony (Table 2). Isolates BB2 and ACCR21 showed phosphate solubilization index ≥3 PSI; while the other six (AE3, AF1, CF1, EA1, EF1, and ACCR4) isolates showed from 2.81 to 2.34 PSI (Table 2).

The HCN-producing ability of all endophytic isolates was tested. Seven (64%) isolates exhibited an orange-brown color of Whatman filter paper no. 1 (soaked in 2% sodium carbonate in 0.1% picric acid solution) that confirmed a positive result and four isolates (AE3, BB2, CF1, and ACCE1) could not produce HCN (Table 1).

In CAS agar plate media, an orange-colored halo zone formed around the colonies, indicating that bacterial strains were producing siderophores. All the bacterial isolates selected in the study produced siderophores, and strains AE2, AF1, and EF1 had the greatest capacity for siderophore production (Table 2). The concentration of siderophore generated by bacterial strains ranged from 06.57 ± 1.59 to 71.23 ± 1.66 PSU. The AF1, EF1, and AE2 isolates displayed the greatest production of siderophores (71.23 ± 1.66, 63.46 ± 1.16, and 59.35 ± 1.19 PSU) among the tested isolates (Table 2).

**FIGURE 2:**
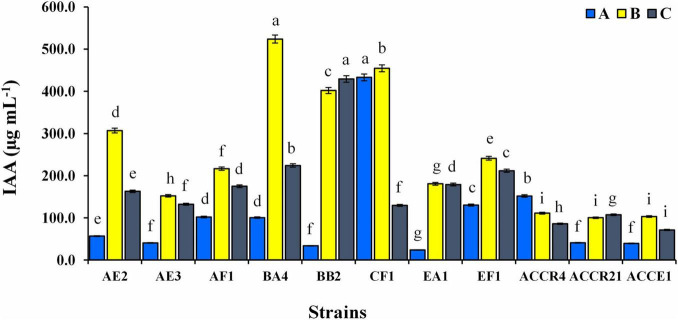
Indole-3-acetic acid production of endophytic bacterial isolates at different concentrations of L-tryptophan (A) absence of L-tryptophan and (B,C) presence of L-tryptophan (0.5 and 1.0 g L^–1^). Dissimilar lowercase letters show a significant difference at *p* ≤ 0.05.

The quantitative estimation of IAA production of all endophytic isolates is shown in Figure 2. In the presence of L-tryptophan (0.5 and 1%), IAA production was higher but it showed considerable variation depending on the amount of tryptophan (100.35 ± 0.75–523.65 ± 9.50 and 71.42 ± 1.30–429.39 ± 7.79 μg mL^–1^). AF1, BA4, CF1, EF1, EA1, and ACCR4 strains displayed significantly higher amounts of IAA production with an increase in tryptophan concentration up to ≥100–432 μg mL^–1^ without addition of L-tryptophan. The maximum IAA production without supplementation of L-tryptophan in the medium (432.94 ± 7.85 μg mL^–1^) was found in CF1 isolate (Figure 2).

Ammonia production is another important PGP feature of PGPEB. This feature was seen in all chosen endophytic isolates that used peptone as a substrate to produce ammonia (Table 2). AE2, AF1, and EF1 isolates produced maximum ammonia, which was 6.18 ± 0.14, 6.33 ± 0.14, and 6.24 ± 0.13 μmoL mL^–1^. Isolates BA4, ACCR21 and BB2, showed in descending rank depending on ammonia production capacity ≥5 moL mL^–1^ (Table 2).

### Biocontrol Activity and Hydrolytic Enzymes Production of Endophytic Bacterial Isolates

**FIGURE 3:**
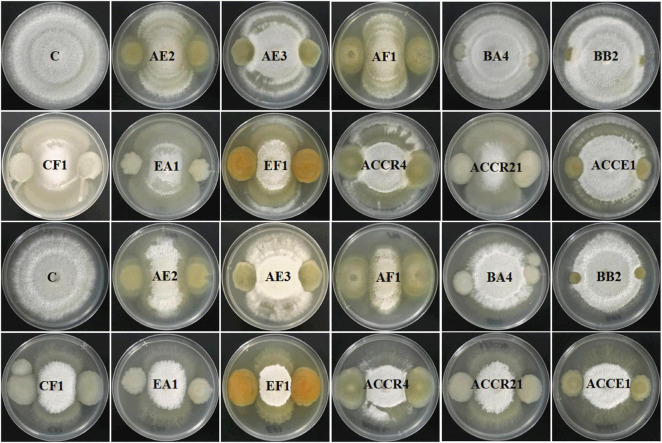
The activity of all selected Gram-negative endophytic antagonistic bacteria against *Fusarium verticillioides* and *Fusarium oxysporum* f. sp. *cubense* pathogens in dual culture and inhibition percentage by culture filtrate method.

The antagonistic potential of 11 identified endophytic strains (*Pantoea dispersa*, *Kosakonia oryzae-* 2, *P. cypripedii*, *P. ananatis, P. agglomerans-* 2, *K. pseudosacchari*, *K. oryziphila, K. arachidis*, and *K. radicincitans*) was examined against two fungal pathogens (*FV* and *FOC*). Results showed that reduction of *FV* mycelia by strains AE2, AF1, and EF1 was more than 55%, whereas AE2, AF1, CF1, EA1, and EF1 strains were able to reduce the growth of *FOC* pathogen. Among all, AF1 and EF1 strains showed (≥ 60%) maximum inhibition of both pathogens (Figure 3).

**FIGURE 4:**
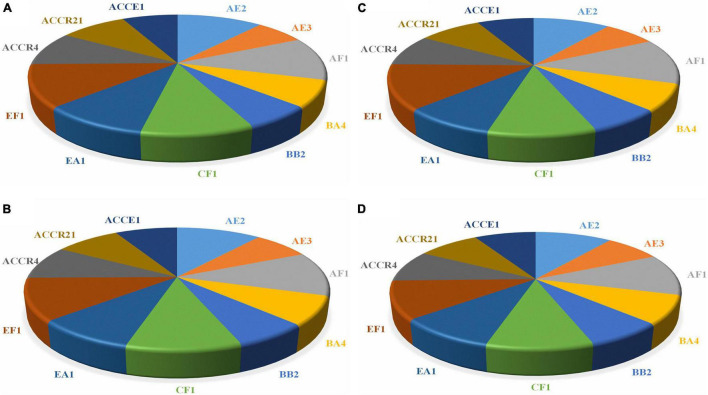
The endophytic isolates displaying different concentrations of hydrolytic enzymes activity and proficient to degrade the cell wall of fungal pathogens. **(A)** Chitinase, **(B)** Protease, **(C)** Cellulase, and **(D)** Endoglucanase.

Figure 4, showing the quantitative estimation of enzymes, i.e., chitinase, protease, cellulase, and endoglucanase of all selected endophytic strains. Endophytic isolates of sugarcane roots were able to generate a large quantity of hydrolytic enzyme activities, which ranged from 386.87–711.37, 137.39–239.85, 741.56–1363.02, and 1530.26–2388.28 IU mL^–1^, respectively. The strain AF1 showed the maximum enzymatic activities of all enzymes as compared to EF1 and other potential strains (Figure 4).

### Scanning Electron Microscopy of Bacteria-Fungal Pathogens Interaction

**FIGURE 5:**
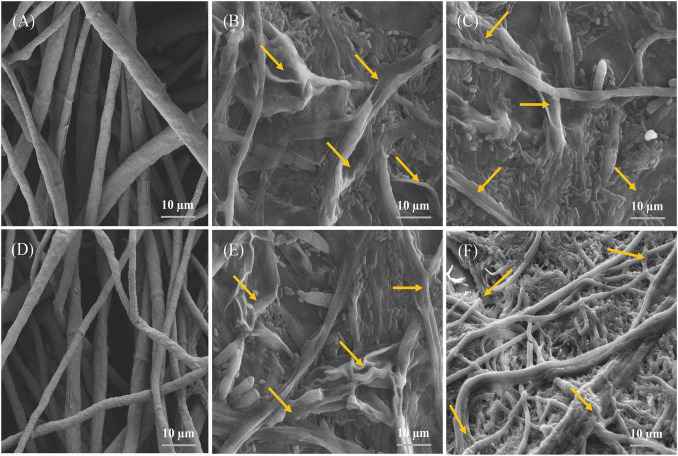
Images obtained by scanning electron microscopy of the antagonistic bacteria interacting with hyphae of selected fungal pathogens on NA: PDA medium after 5 days incubation. **(A,D)** indicating a normal hyphae of *F. verticillioides* and *F. oxysporum* f. sp. *cubense*, whereas **(B,C,E,F)**, indicating abnormal hyphae of selected fungal pathogens.

Inhibition of both pathogens’ growth by AF1 and EF1 isolates was further validated *in vitro* by SEM investigations (Figure 5). Control plates- without strains AF1 and EF1 had healthy mycelia of *FV* and *FOC* pathogens, i.e., with regular in shape and cylindrical (Figures 5A,D), whereas, morphological changes were observed in the mycelia of *FV* and *FOC* inoculated with pathogens AF1 and EF1 isolates had broken mycelial surface and fragmentation of mycelia (Figures 5B,C,E,F).

### Effect of Abiotic Stress Factors

**FIGURE 6:**
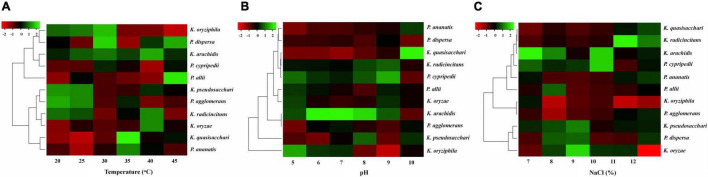
Effects of different abiotic stresses on selected isolates. **(A)** Temperature, **(B)** pH level, and **(C)** Different concentrations of salt.

In this study, growth was measured for all isolates in various abiotic stress environments, i.e., temperature (20–45°C), pH (5–10), and NaCl (7–12%) and the results are presented using a heatmap (Figure 6).

All isolates showed maximum favorable growth at 25–35°C. However, at 40°C the growth of the isolates was decreased, and at 45°C growth was observed but decreased remarkably with only a few isolates showed little growth, as shown in Figure 6A. The result of diverse pH values (5–10) on the growth of all diazotrophs is shown in Figure 6B. All diazotrophs showed little growth at pH 5 but grew well from pH 6 to 9. Also, it was observed that all isolates showed optimum growth up to 7–8% NaCl concentration, but increasing NaCl concentration beyond that level (9–12% NaCl) decreased growth, and only a few strains showed very limited growth at 12% NaCl (Figure 6C).

### Localization of Diazotrophs in the Sugarcane Plant

**FIGURE 7:**
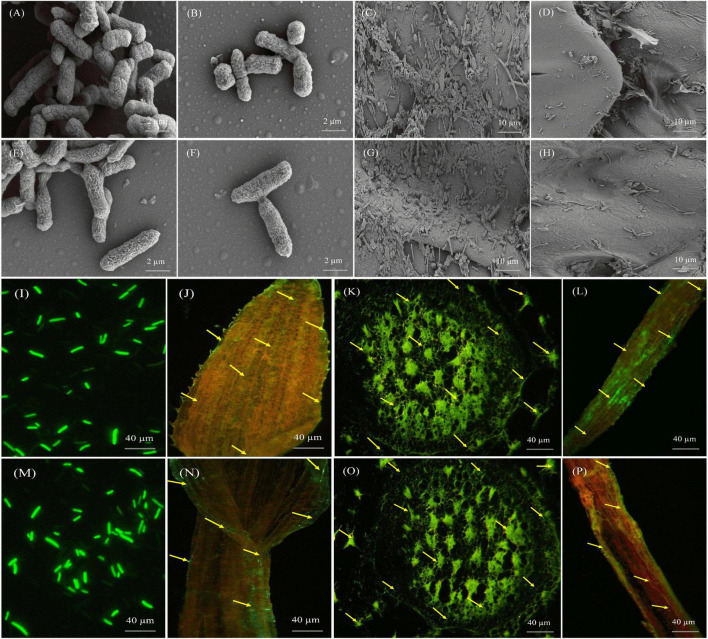
Microscopic pictures presenting the morphology and colonization of *Pantoea cypripedii* AF1 and *Kosakonia arachidis* EF1 in sugarcane (GT42). **(A,B,E,F)** Scanning electron micrographs (SEM) displaying morphology (rod-shaped) of AF1 and EF1 bacteria. **(C,D,G,H)** Colonization of AF1 and EF1 in root and stem tissues of sugarcane plant. **(I,M)** Confocal laser scanning micrographs (CLSM) displaying morphology of GFP tagged AF1 and EF1 strains. **(J–L,N–P)** Colonization of AF1 and EF1 strains as green dots in leaf, stem, and root tissues of sugarcane.

Scanning electron microscopy was also used to confirm the colony morphology and colonization of AF1 and EF1 in the root and stem of sugarcane plants (Figures 7A–H). In general, inoculated plants showed the colonization of bacterial isolates on the root and stem with high density and it was dispersed throughout the plant body. This result suggests that both diazotrophic isolates are aggressive endophytic colonizers of the sugarcane cultivated regions.

We also examined the colonization of AF1 and EF1isolates by CLSM in sugarcane plant tissues (root, stem, and leaf). Figure 7 clearly shows the colonies of bacterial isolates inside the root, stem, and leaf tissues. Both isolates colonized root hairs and showed their presence in root epidermal cells when inoculated separately. Green fluorescence of GFP-tagged bacterial isolates was detected in numerous cells in different tissues throughout the plant (Figure 7). At 40× resolution, GFP-tagged cells were localized in inter-cellular regions, within the vascular tissues, and in fissures at the points of lateral root emergence (Figures 7J–L,N–P).

### Studies on N Metabolism and Pathogen-Control Related Genes

**FIGURE 8:**
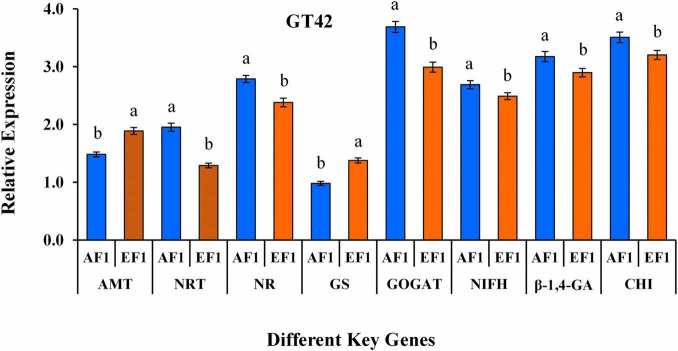
qRT-PCR study of aminomethyl transferase*- AMT*, nitrate transporter*- NRT*, nitrate reductases- *NR*, glutamate synthase- *GS*, glutamine synthetase- *GOGAT*, nitrogen fixation- *nifH*, endo-glucanase- β*-1,4-GA*, and chitinase- *CHI* genes in root tissue of sugarcane variety (GT42) inoculated with *P. cypripedii* AF1 and *K. arachidis* EF1 strains. The GAPDH expression level was used to standardize the data. All data points are viewed as the mean ± SE (*n* = 3). Different letters specify significant differences among the treatments at *p* ≤ 0.05.

RT-qPCR was used to quantify the expression level of N metabolism (*AMT*, *NRT*, *NR*, *GS*, *GOGAT*, and *NifH*) and biocontrol (β*-1,4-GA*, and *CHI*) related genes in root tissues of sugarcane plant (Variety- GT42) after AE1 and EF1 inoculation (Figure 8). The results showed significant changes in the expression of all the analyzed genes in sugarcane after AE1 and EF1 inoculation as compared to control. Whereas AF1-inoculated plants showed higher expression levels of *NRT*, *NR*, *GOGAT*, *NifH*, β*-1,4-GA*, and *CHI* genes, and expression of *AMT* and *GS* was maximum in EF1inoculated plants (Figure 8).

### Biological Nitrogen Fixation Contribution by the ^15^N Isotopic Dilution Method

**FIGURE 9:**
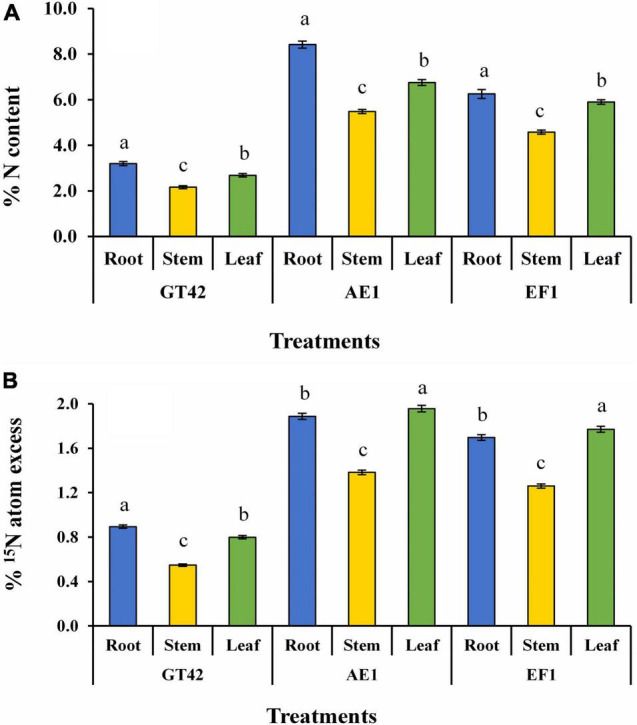
Estimation of biological nitrogen fixation in sugarcane tissues (dry matter yield of root, stem, and leaf) by *P. cypripedii* AF1 and *K. arachidis* EF1 cultivated in ^15^N-labeled soil (values are the average of three repeats) **(A)** % N content and **(B)** % ^15^N atom excess. Significant changes among treatments at *p* ≤ 0.05 are showed by dissimilar letters.

^15^N isotope dilution method showed that N content was higher in sugarcane plants (GT42) after inoculation of AE1 and EF1 strains as compared to control plants (Figure 9). The maximum tissue concentration of N was found in roots than leaf and stem tissues, respectively (Figure 9A). Whereas, % ^15^N atom excess of sugarcane leaf was higher than the root and stem tissues after bacterial inoculation (Figure 9B). Therefore, biological N fixation in sugarcane by selected isolates was confirmed.

### Greenhouse Assay for Diazotrophic Isolates

We measured the different physiological growth parameters such as plant height, root and shoot weight, leaf area, chlorophyll content, photosynthesis, transpiration rate, stomatal conductance, and total protein content of sugarcane (GT42) inoculated with (AF1 and EF1) strains under greenhouse conditions. As compared to control, isolates AF1 and EF1 showed increased plant height, root weight, and shoot weight, i.e., 46, 32, and 72% and 31, 169, and 123%, respectively (Table 3). Whereas, no more significant difference was observed in leaf area and total protein content of sugarcane (Table 3). Table 3 shows significantly higher chlorophyll content (SPAD values) in inoculated sugarcane leaves than in control plants. In general, the SPAD values were lower in EF1 than AF1. The photosynthesis rate was marginally higher in plants-inoculated with AF1 (19.82 ± 0.19 μ mol CO_2_ m^–2^ s^–1^) than those with EF1 (18.55 ± 0.18 μ mol CO_2_ m^–2^ s^–1^). And, it was also found that the transpiration rate in the sugarcane plant was significantly lower in EF1 than AF1 inoculated bacteria but as compared to the control plant both isolates increased up to 131 and 120% (Table 3). When comparing both strain’s stomatal conductance of sugarcane plants, there were no more significant differences observed, but over control, the observed percent increase values were 107 and 105 for AF1 and EF1 strains (Table 3). Overall, these results suggest that both selected diazotrophic isolates are effective in sugarcane growth enhancement, and the application of strain AF1 showed more significant improvement as compared to EF1 in GT42.

**TABLE 3:** The assessing of diazotrophic endophytes *Pantoea cypripedii* AF1 and *Kosakonia arachidis* EF1 inoculum on the sugarcane (variety GT42) in the greenhouse experiment.

Parameters	Treatments	Tillering stage	% Change over control
Plant height (cm)	GT42	36.55 ± 0.35^c^	–
	AF1	53.39 ± 0.50^a^	46.08
	EF1	48.31 ± 0.46^b^	32.18
Root weight (g)	GT42	3.26 ± 0.03^c^	–
	AF1	5.63 ± 0.05^a^	72.70
	EF1	4.28 ± 0.04^b^	31.29
Shoot weight (g)	GT42	32.38 ± 0.31^c^	–
	AF1	87.25 ± 0.82^a^	169.46
	EF1	72.46 ± 0.68^b^	123.78
Leaf area (cm^2^)	GT42	204.46 ± 1.93^c^	–
	AF1	448.39 ± 4.24^b^	119.30
	EF1	465.11 ± 4.39^a^	127.48
Leaf length (cm)	GT42	114.24 ± 5.73^b^	–
	AF1	221.85 ± 3.34^a^	94.20
	EF1	208.80 ± 9.30^a^	82.77
Leaf width (cm)	GT42	2.29 ± 0.14^b^	–
	AF1	2.83 ± 0.11^a^	23.44
	EF1	2.66 ± 0.10^a^	16.16
Plant diameter (mm)	GT42	10.28 ± 0.44^c^	–
	AF1	13.69 ± 0.52^a^	33.20
	EF1	12.32 ± 0.48^b^	19.84
Total protein (μg g^–1^ Fresh weight)	GT42	1337.50 ± 12.64^b^	–
	AF1	1755.77 ± 16.59^a^	31.27
	EF1	1783.23 ± 16.85^a^	33.33
Chlorophyll content (SPAD units)	GT42	33.80 ± 0.32^c^	–
	AF1	43.42 ± 0.41^a^	28.47
	EF1	41.44 ± 0.39^b^	22.62
Photosynthesis (μ mol CO_2_ m^–2^ s^–1^)	GT42	10.38 ± 0.10^c^	–
	AF1	19.82 ± 0.19^a^	90.97
	EF1	18.55 ± 0.18^b^	78.72
Transpiration rate (mmol H_2_O m^–2^ s^–1^)	GT42	1.14 ± 0.01^c^	–
	AF1	2.64 ± 0.02^a^	131.80
	EF1	2.52 ± 0.02^b^	120.84
Stomatal conductance (mmol H_2_O m^–2^ s^–1^)	GT42	39.63 ± 0.37^b^	–
	AF1	82.42 ± 0.78^a^	107.97
	EF1	81.31 ± 0.77^a^	105.18

*Different alphabets indicate significant differences between treatments at p ≤ 0.05.*

### Measurement of N Metabolism and Biocontrol Enzymes Activities

**FIGURE 10:**
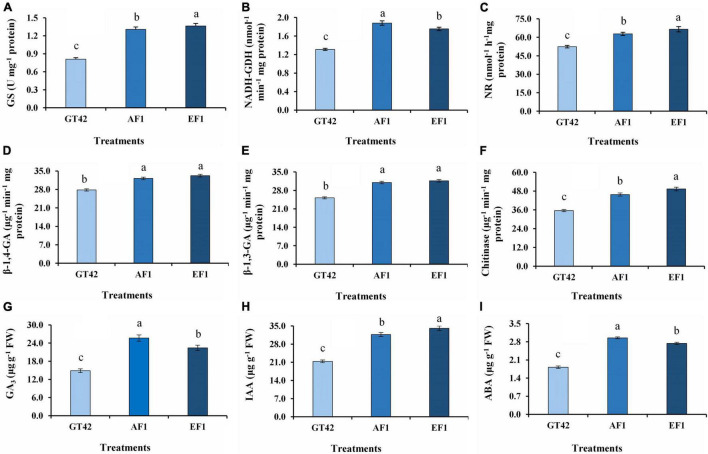
Analysis of N-metabolism enzymes, hydrolytic enzymes, and hormone activities in root tissues of sugarcane (GT42) inoculated with *P. cypripedii* AF1 and *K. arachidis* EF1. **(A)** Glutamine synthetase, **(B)** glutamate dehydrogenase, **(C)** nitrate reductase, **(D,E)** β-1,4 and β-1,3 glucanase, **(F)** chitinase, **(G)** gibberellins, **(H)** indole-3-acetic acid, and **(I)** abscisic acid. Different lowercase letters display a significant difference at *p* ≤ 0.05.

An increased level of selected N metabolism and biocontrol-related enzymes was observed in sugarcane plants (GT42) treated with diazotrophs (AF1 and EF1) (Figure 10). The plants inoculated with AF1 and EF1 showed 61 and 68% greater GS activity than control plants (Figure 10A), whereas, increase in NADH-GDH content was higher in AF1 (43%) inoculated plants than EF1 (36%) as compared to their controls (Figure 10B). Following inoculation with isolates, NR activity was higher in EF1 and AF1 inoculated plants (66.56 ± 2.11 and 62.78 ± 1.33 n mol^–1^ h^–1^mg proteins) than control (52.39 ± 0.98 n mol^–1^ h^–1^ mg proteins) (Figure 10C). Inoculation with endophytic diazotroph EF1 and AF1 significantly enhanced 19 and 16% β-1,4-glucanase activity in the root as compared to non-inoculated sugarcane plants (Figure 10D). A similar pattern of β-1,3-glucanase activity was observed in sugarcane inoculated with both isolates (AF1 and EF1) (Figure 10E). A significant increase in chitinase activity (28 and 38%) was observed in roots of GT42 inoculated with AF1and EF1 as compared to non-inoculated sugarcane plants (Figure 10F).

In this study, the significant difference in GA_3_, IAA, and ABA level was observed between the roots of bacteria-inoculated and uninoculated sugarcane plants (Figure 10). The treatment of plants with AF1 and EF1 increased GA production in root by 72 and 51%, respectively, as compared to control (Figure 10G). The IAA production in the root was increased more with the EF1 inoculation (Figure 10H). EF1 and AF1 inoculation increased IAA content by 59 and 48%, respectively, in inoculated plants, as compared to the non-inoculated plants. ABA production by sugarcane plants inoculated with AF1 and EF1 strains in 62 and 50%, respectively, higher compared with the non-inoculated plants.

## Discussion

Diazotrophs, known for their BNF properties, also produce antibiotics, HCN, and siderophores, as well as build systemic resistance in plants, to inhibit a wide spectrum of plant fungal pathogens (Das et al., 2017; Ali et al., 2020). Hence, the development of diazotrophic bacteria as a biofertilizer with multi-functional traits might help boost plant growth and crop production. In a previous survey of sugarcane-producing regions in Guangxi, China, Lin et al. (2012) found that *Klebsiella* is the most abundant plant-associated N-fixing bacteria. Thus, the screening and selection of different diazotrophic bacteria with PGP activities will be valuable for sustainable sugarcane agriculture, especially in low fertile soils. In this research, a culture-independent approach was used to discover the potential Gram-negative endophytic diazotrophic bacteria along with PGP features for their potential application as biofertilizers in sugarcane. A total of 175 endophytic isolates were isolated and after 16S *rRNA* gene sequencing, we selected 11 strains of genus *Kosakonia* (*K. oryzae*, *K. radicincitans*, *K. arachidis*, *K. oryziphila*, *K. quasisacchari*, and *K. pseudosacchari*) and *Pantoea* (*P. dispersa*, *P. cypripedii*, *P. ananatis*, *P. allii*, and *P. agglomerans*). A number of different diazotrophic bacterial species isolated from sugarcane can also be opportunistic pathogens, but these microbes usually do not cause diseases in a healthy host (Fishman, 2013; Berg et al., 2014). The strains selected in this study also showed abiotic stress tolerance properties as they grew on a wide range of temperatures (20–45°C), pH (5–10), and salinity (7–12% NaCl), suggesting that these isolates may tolerate stressful crop production environments. Two strains *P. cypripedii* AF1 and *K. arachidis* EF1, which exhibited multiple PGP traits, were selected for detailed study.

The use of N-fixing beneficial microbes in agriculture might reduce the usage of chemical N fertilizers in agriculture, and reducing their negative environmental impacts (Noar and Bruno-Bárcena, 2018). Both selected strains showed the presence of *nifH* gene, which encodes the nitrogenase reductase enzyme. We observed the enhanced expression of N metabolism (*AMT, NRT*, *NR, GS*, and *GOGAT*) and BNF-related genes (*nifH*) in sugarcane after inoculation with AF1 or EF1 strains. *AMT* and *NRT* facilitate nitrate-N and ammonium-N uptake in the roots (Singh et al., 2016). The expression of *NRT, NR*, and *GOGAT* was higher in EF1-inoculated GT42 roots compared with AF1-inoculated ones with, indicating the strain variability for plant N metabolism regulation. Nitrate is taken up by roots and converted into glutamine and glutamate, which are used by plants to make other amino acids and nitrogenous compounds (Pratelli and Pilot, 2014). The primary assimilation of nitrate-N and ammonium-N in plants is mediated by two important N-assimilation enzymes, NR and GS (Funayama et al., 2013). The activities of N metabolism-related enzymes (GS, NADH-GDH, and NR) in AF1- and EF1-inoculated plants show that these bacteria had considerably increased N use efficiency in sugarcane (Figure 10). Glutamate synthase is a vital enzyme in N assimilation, metabolism, and remobilization, and its activity is influenced by environmental factors (Miflin and Habash, 2002; Stitt et al., 2002). It was observed that the activity of GS was higher in EF1 than that observed for AF1 (Figure 10). The high concentration of GS1 transcripts in sugarcane leaves implies that this isoform plays an important role in C4 plants for N metabolism (Nogueira et al., 2005). Ammonia production by PGP diazotrophic isolates contributes N to the host plants and supports biomass production (Marques et al., 2010). Our results showed that all Gram-negative endophytic bacteria produced ammonia, with *P. cypripedii* AF1 and *K. arachidis* EF1 being more efficient than the other isolates.

The technique of ^15^N isotope dilution has been frequently utilized to measure the contribution of associative N-fixing systems to graminaceous crops like sugarcane (Urquiaga et al., 1992; Asis et al., 2002; Montañez et al., 2009; Thaweenut et al., 2011; Lin et al., 2012; Li et al., 2017; Singh et al., 2020c,2021b). This study was performed in pots that used total N balance and enriched ^15^N fertilizer isotope-dilution methods, and the outcome was impressive. The findings of the ^15^N enrichment indicated that there was a significant amount of non-labeled N present, which could only have originated from BNF (Martins et al., 2020). Previous, investigations have found that Brazilian sugarcane cultivars were able to acquire 40–100 kg N ha^–1^ yr^–1^ from BNF utilizing plants without any treatment. A significant development of plant growth and N accumulation using diazotrophic bacteria was also observed in different sugarcane varieties (Li et al., 2017; Singh et al., 2020b). However, even after three decades of research, our understanding as to how the diazotrophs work, fix N, and transfer the fixed N to the crop remains limited (Martins et al., 2020).

Plant growth and crop productivity can be improved by siderophore-producing diazotrophic bacteria by improving Fe accessibility and boosting plant growth (Ahmed and Holmström, 2014). As expected, all chosen strains were capable of generating iron-chelating siderophores at various levels, and this trait is usually present in *Pantoea* and *Kosakonia* genera (Chen et al., 2017; Lambrese et al., 2018; Romano et al., 2020). Also, in this study, only eight diazotrophic isolates (73%) were able to solubilize phosphate. In the soil, Phosphate (P) is present abundantly in most a crop land but mostly unavailable to the plants, hence this is one of the main growth-limiting nutrients for plants in agricultural systems. It’s also critical for root formation, early shoot growth and tillering, early productivity, and plant stem elongation. Previously, a few strains of genus *Kosakonia* with P solubilizing ability were reported (Chakdar et al., 2018; Singh et al., 2020b). And, Chen and Liu (2019), studies found the genus *Pantoea* a highly effective P-solubilizing bacterium.

Hydrogen cyanide is a volatile secondary metabolite generated by several endophytic bacterial isolates involved in disease suppression and protection of the plants, particularly from fungal pathogens (Olanrewaju et al., 2017). The results showed that only 63% of endophytic isolates produced HCN. Strains that tested positive for HCN production were shown to be effective against sugarcane pathogens in this study. In many respects, these PGP results are similar to previous studies (Li et al., 2017; Guo et al., 2020; Singh et al., 2021a).

The sugarcane plant is affected by several fungal pathogens, therefore we screened 11 endophytic isolates for the antifungal property. Among them two selected strains showed good antagonistic activity against fungal pathogens, which was further corroborated by the degradation of fungal mycelia seen during SEM analysis. This finding supports earlier observations that, some diazotrophic bacterial strains isolated from sugarcane can suppress the plant fungal pathogens (Li et al., 2017; Guo et al., 2020). Additionally, all isolates were also tested for an important role in the production of hydrolytic enzymes such as chitinase, protease, cellulase, and endoglucanase. The role of these enzymes in biological control of various fungal pathogens has been previously reported (Huang et al., 2005; Singh et al., 2013; Passari et al., 2017, 2018). Pot experiment also showed the interaction of *P. cypripedii* AF1 and *K. arachidis* EF1 strains enhanced the production of CHI and GLU in root tissues of sugarcane. Similarly, CHI and GLU produced by different strains of genera *Pantoea* and *Kosakonia* improved sugarcane growth (Quecine et al., 2012; Singh et al., 2021b). The colonization of endophytic bacteria on plant tissues is also playing a crucial role in disease management and plant growth enhancement (Li et al., 2017; Guo et al., 2020). We examined the colonization of selected endophytic strains individually with genetically tagged GFP and found that both strains effectively colonized all plant tissues.

Our findings revealed a substantial influence of diazotrophic bacterial inoculation of sugarcane plant (GT42) with GA_3_, IAA, and ABA content. Sugarcane inoculated with selected isolates had considerably greater levels of all phytohormones than GT42 control. These hormones promote plant growth, root development, fertilizer absorption and assimilation, water uptake and a variety of metabolic processes in response to abiotic and biotic stresses (Graham, 2003; Stepanova et al., 2007; Singh et al., 2019). 1-aminocyclopropane-1-carboxylate deaminase activity is one of the principal mechanisms of endophytic bacteria that can assist plant growth in the presence of various biotic and abiotic stresses. All endophytic isolates selected in this study synthesized ACC deaminase, and the presence of *acdS* gene was confirmed in eight isolates. The reason for the inability to detect this gene in three isolates remains unclear. Endophytic bacteria protect host plants from a range of stress conditions by generating chemicals and enzymes such as ACC deaminase to decrease ethylene production (Glick, 2014; Mercado-Blanco and Lugtenberg, 2014). Apart from conferring stress tolerance, ACC deaminase also promotes root growth, which will have a positive effect on healthy shoot development (Belimov et al., 2002; Glick et al., 2007). Bacterial isolates with ACC deaminase activity have been reported, but it did not show the presence of *acdS* gene (Nascimento et al., 2014).

## Conclusion

Despite the variety of bacterial species identified in sugarcane-producing habitats, there is little information regarding the diversity of Gram-negative bacteria, their ecology, and biotechnological potential in commercial sugarcane production. The significance of this study is highlighted by the promotion of plant growth and development by the Gram-negative endophytic diazotrophs *P. cypripedii* AF1 and *K. arachidis* EF1, and this is the first report to show that these two diazotrophic bacteria reside in sugarcane roots and potentially contributing to sugarcane growth, development, and disease suppression. These isolates exhibited a variety of PGP characteristics, including antifungal action against plant pathogens, N fixation, and the synthesis of enzymes and phytohormones. Further research into these diazotrophic isolates is needed to evaluate their commercial value as bio-fertilizers for improved sugarcane agricultural productivity.

## Data Availability Statement

The datasets presented in this study can be found in online repositories. The names of the repository/repositories and accession number(s) can be found in the article/Supplementary Material.

## Author Contributions

RS, PS, and Y-RL designed the experiments. RS, PS, and D-JG conducted the majority of the experiments. RS and PS wrote the article. PL, L-TY, and Y-RL reviewed and editing the article. The other authors assisted in experiments and discussed the results. All authors read and approved this manuscript.

## Conflict of Interest

The authors declare that the research was conducted in the absence of any commercial or financial relationships that could be construed as a potential conflict of interest.

## Publisher’s Note

All claims expressed in this article are solely those of the authors and do not necessarily represent those of their affiliated organizations, or those of the publisher, the editors and the reviewers. Any product that may be evaluated in this article, or claim that may be made by its manufacturer, is not guaranteed or endorsed by the publisher.
