# Supplementary material for: Root-Derived Endophytic Diazotrophic Bacteria Pantoea cypripedii AF1 and Kosakonia arachidis EF1 Promote Nitrogen Assimilation and Growth in Sugarcane
Source: Front Microbiol. 2021 Dec 15;12:774707. doi: 10.3389/fmicb.2021.774707 (PMC8714890; doi:10.3389/fmicb.2021.774707)
Supplement: Supplementary file 1 [file Data_Sheet_1.docx]

Supplementary Material

# Supplementary Tables

**Table S1.** Different medium used in this study for isolation and screening of endophytic diazotrophs from sugarcane.

| **S. No.** | **Medium name and composition (g L^-1^)** |
| --- | --- |
| **Ashbey’s Medium** | Mannitol; 15, CaCl_2_.2H_2_O; 0.2, MgSO_4_.7H_2_O; 0.2, MoO_3_ (10% solution); 0.1 mL, FeCl_3_ (10% solution); 0.05 mL, Agar; 15 |
| **Yeast Mannitol Agar Medium** | Mannitol; 15, K_2_HPO_4_; 0.5, Yeast Extract; 0.4, MgSO_4_.7H_2_O; 0.2, NaCl; 0.1, Agar; 15 |
| **JNFb medium (****Baldani et al. 1992)** | Malic acid, 5.0; K_2_HPO_4_, 0.6; KH_2_PO_4_, 1.8; MgSO_4_.7H_2_O, 0.2; NaCl, 0.1; CaCl_2_.2H_2_O, 0.02.  Micronutrient solution: (CuSO_4_.5H_2_O, 0.04; ZnSO_4_.7H_2_O, 0.12; H_3_BO_3_, 1.40; Na_2_MoO_4_.2H_2_O, 1.0; MnSO_4_. H_2_O, 1.175) (g L^-1^).  2 mL; bromothymol blue (5 g L^−1^ in 0.2 N KOH), 2 mL; Fe EDTA (16.4 g L^−1^), 4 mL; vitamin solution (biotin, 10 mg; pyridoxal-HCl, 20 mg, dissolved in a hot-water bath. Complete to 100 mL by adding distilled water), 1 mL; KOH, 4.5g. Add distilled water to bring the total solution to 1,000 mL. Adjust the pH to 6.8 with KOH. |
| **LGI Medium** | Sucrose; 5, KH_2_PO_4_; 0.6; K_2_HPO_4_; 0.2; MgSO_4_.7H_2_O; 0.2, CaCl_2_.2H_2_O; 0.02, FeCl_3_; 1, Na_2_MoO_4_. 2H_2_O; 2 mg, Bromothymol Blue Solution; 5 mL, Agar; 1.75 |
| **Nutrient Agar** | Peptone; 5, NaCl; 5, Yeast Extract; 2, Beef Extract; 1, Agar; 15 |
| **Potato Dextrose Agar** | Potatoes (infusion from); 200, Dextrose; 20, Agar; 15 |
| **DF salts minimal medium** | Glucose; 2, Gluconic acid; 2, Citric acid; 2, KH_2_PO_4_; 4, Na_2_HPO_4_; 6, MgSO_4_**.**7H_2_O; 0.2, Micro nutrient solution (in mg) (CaCl_2_; 200, FeSO_4_.7H_2_O; 200, H_3_BO_3_; 15, ZnSO_4_.7H_2_O; 20, Na_2_MoO_4_; 10, KI; 10, NaBr; 10, MnCl_2_; 10, COCl_2_; 5, CuCl_2_; 5, AlCl_3_; 2, NiSO_4_; 2. |
| **Luria Bertani Broth** | Casein enzymic hydrolysate; 10, Yeast extract; 5, Sodium chloride; 5. |

**Table S2.** Primer’s sequence used for *16S, nifH, scdS* and *HCN* genes amplification and N-metabolism gene expressions.

| **Gene** | **Primer** | **Sequence (5**′ **-------→ 3′)** | **Reference** |
| --- | --- | --- | --- |
| *16S* | pA-F  pH-R | AGAGTTTGATCCTGGCTCAG  AAGGAGGTGATCCAGCCGCA | Edwards et al., 1989 |
| *nifH* | PolF  PolR | TGCGAYCC-SAARGCBGACTC  ATSGCCATCATYTCRCCGGA | [Poly](http://www.sciencedirect.com/science/article/pii/S0923250800011724) et al., 2001 |
| *acdS* | ACD-F  ACD-R | GCAACAAGACGCGCAAGYTNGARTAYNT  GTGCATCGACTTGCCCTCRWANACNGGRT | Li et al., 2011 |
| *HCN* | HCN-F  HCN-R | ACTGCCAGGGGCGGATGTGC  ACGATGTGCTCGGCGTAC | Ramette et al., 2003 |
| **qRT-PCR primer** | | | |
| *AMT* | AMT-F  AMT-R | CCAAGAACACCATGAACATC  GAAGCCGAAGAGGTAGTAGA | Yang et al., 2019 |
| *NRT* | NRT-F  NRT-R | ACCGTCACCAACTTCATC  AGATCGTCAGGATCATCAC | Yang et al., 2019 |
| *NR* | NR-F  NR-R | CAAGCACATCTTCGTCTG  TGCTCGTTCTTGAAGTAGA | Yang et al., 2019 |
| *GS* | *GS-F*  *GS-R* | GTGTGGTGTTGACATTCG  ATGGACTTGGTGCTGTAG | Yang et al., 2019 |
| *GOGAT* | *GOGAT-F*  *GOGAT-R* | GCAATGAATAGAATAGGTGGAA  ATGAGGAAGTGTTGGAGAATA | Yang et al., 2019 |
| *NifH* | *NifH-F*  *NifH-R* | TGCGAYCCSAARGCBGACTC  ATSGCCATCATYTCRCCGGA | Poly et al., 2001 |
| *β-1,4-GA* | *β-1,4-GA-F*  *β-1,4-GA-R* | TGCTACTTCTTATCCACCCTCTG CGTTGACATAGAAAGGTGAGCC | Su et al., 2013 |
| *CHI* | *CHI-F*  *CHI-R* | ACGGCTACGGCGACAACA GTCCGCTGACCAGATGAAGAG | Su et al., 2014 |
| *GDH* | *GDH-F*  *GDH-R* | TGGTGGTTCATTAGGTAGG  CCGTACTCAGCAAGAAGA | Iskandar et al., 2004 |

*Aminomethyl transferase- AMT, Nitrate transporter- NRT, Nitrate reductases- NR, Glutamate synthase- GS, Glutamine synthetase- GOGAT, Nitrogen fixation- NifH, Endo-glucanase- β-1,4-GA and Chitinase- CHI, Glyceraldehyde 3-phosphate dehydrogenase, GDH*

## Supplementary Figures


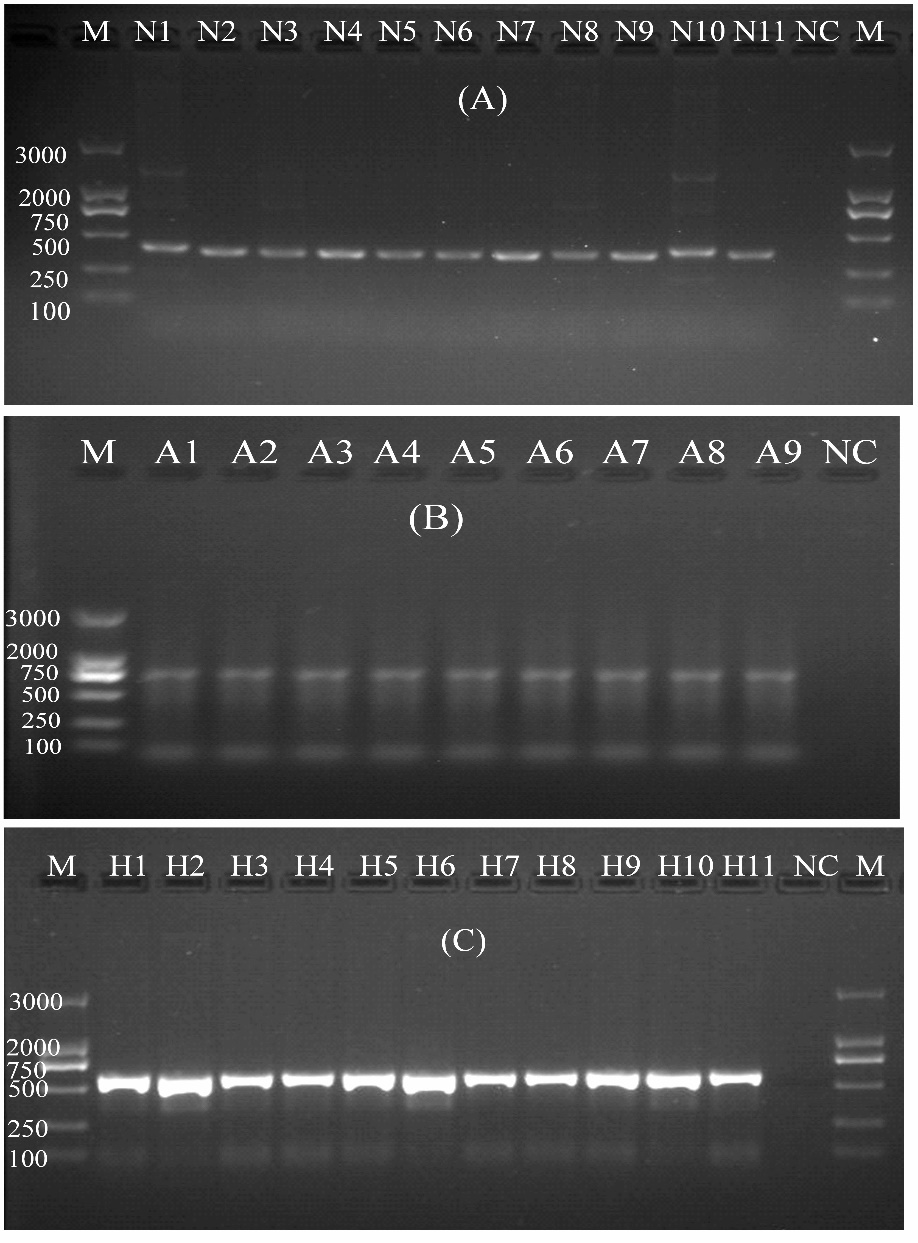


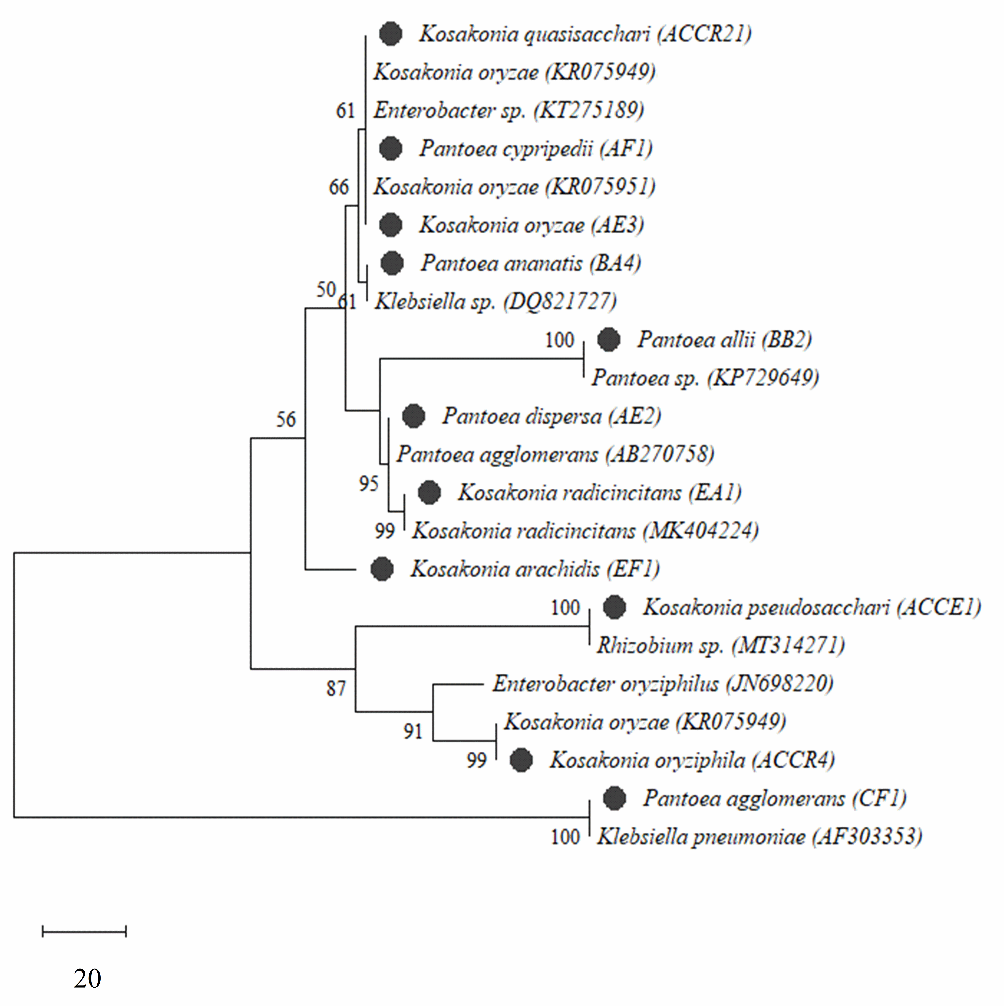


**Figure S1.** PCR amplification of *nifH*, *acdS*, and HCN genes in diazotrophic endophytic bacteria (A-C). M: molecular size marker (100 to 2,000 bp), PC: positive control, and NC: negative control (sterile water) and *nifH* phylogenetic analysis (D).


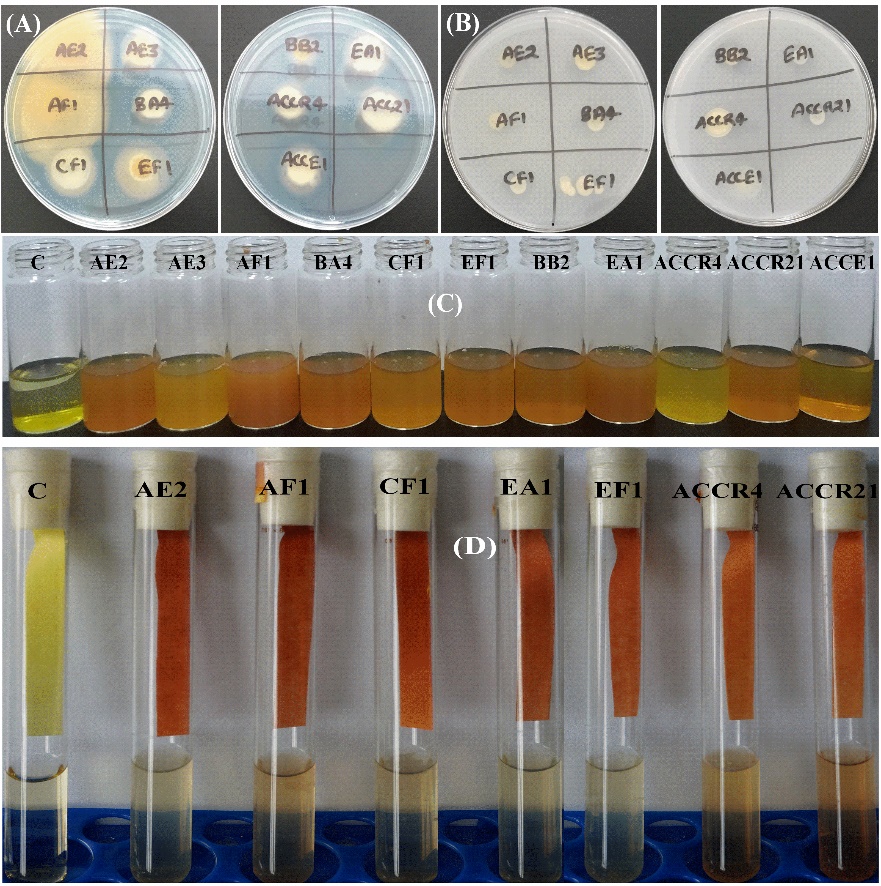


**Figure S2.** Images obtained by *in vitro* screening of different plant growth promoting traits for all endophytic isolates isolated from sugarcane root. (A) Siderophore production, (B) Phosphate solubilization (C) Ammonia production, and (D) Hydrogen cyanide production.

**
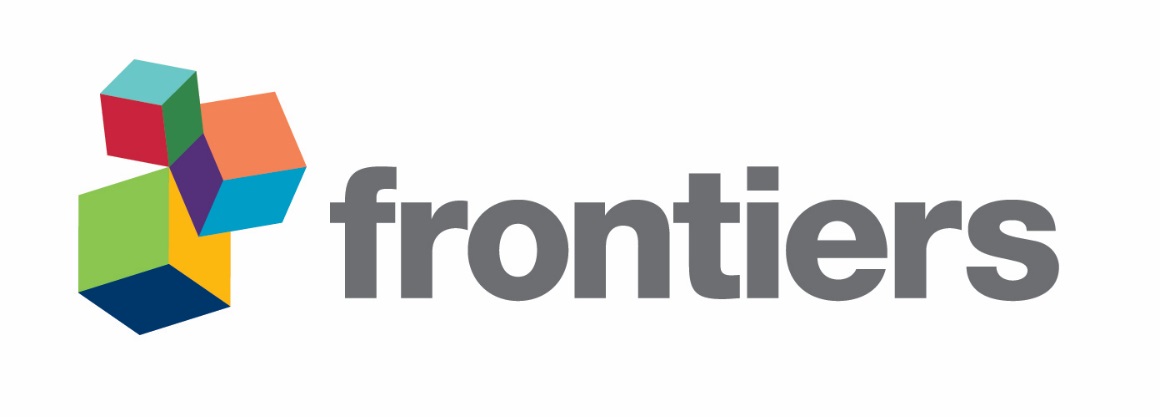
**
