# Supplementary figures and images for: Root-Derived Endophytic Diazotrophic Bacteria Pantoea cypripedii AF1 and Kosakonia arachidis EF1 Promote Nitrogen Assimilation and Growth in Sugarcane
Source: Front Microbiol. 2021 Dec 15;12:774707. doi: 10.3389/fmicb.2021.774707 (PMC8714890; doi:10.3389/fmicb.2021.774707)

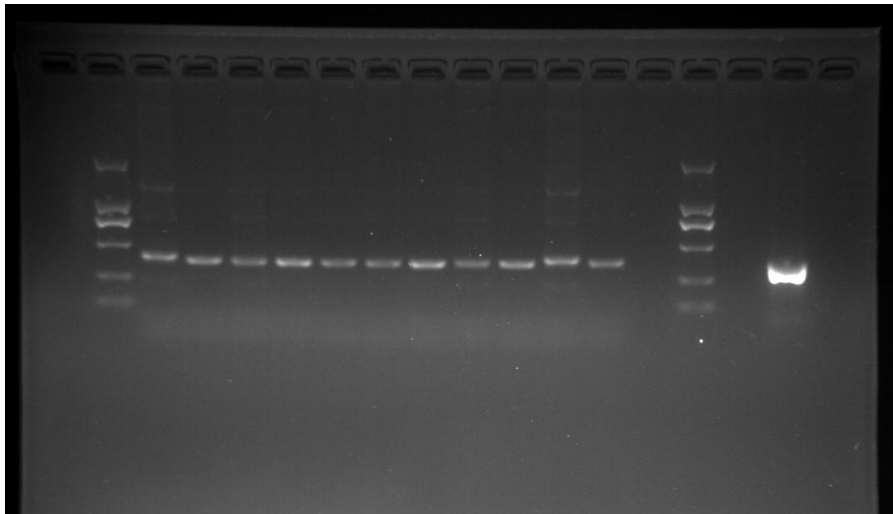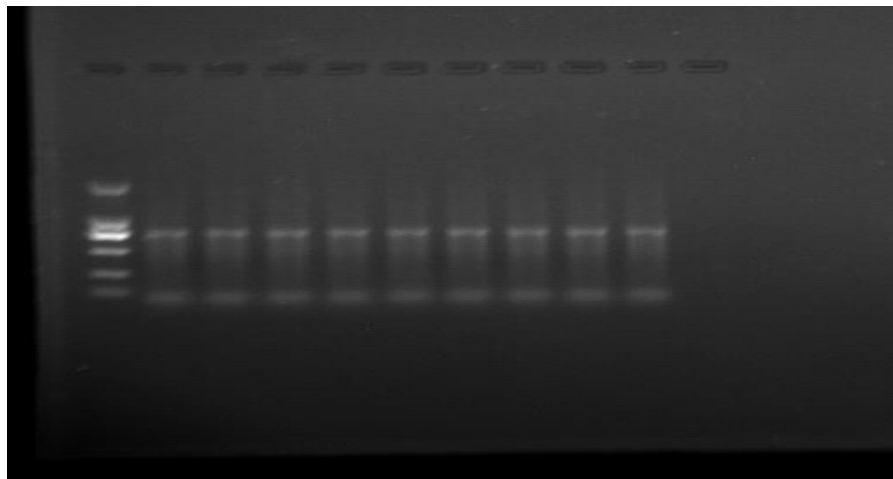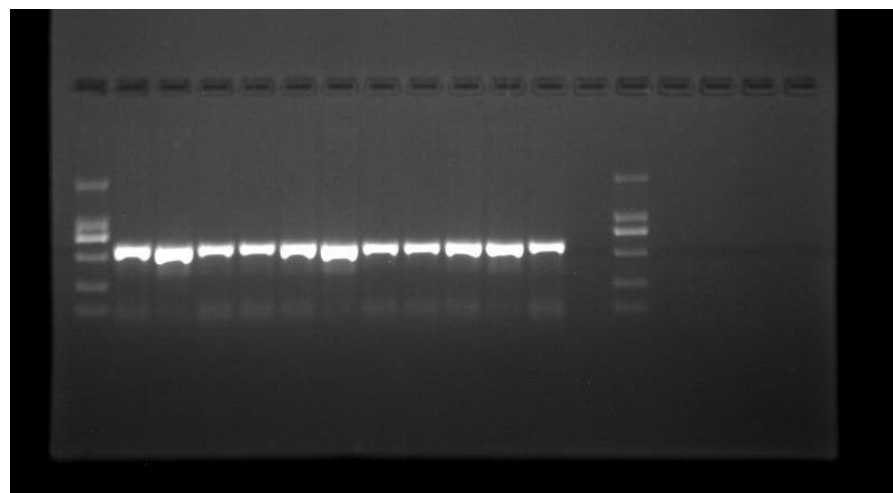

**Figure S1.**

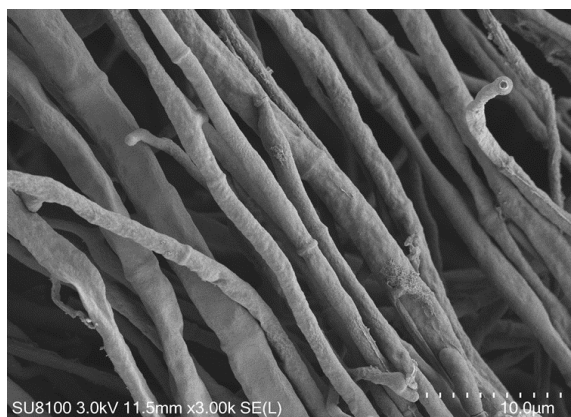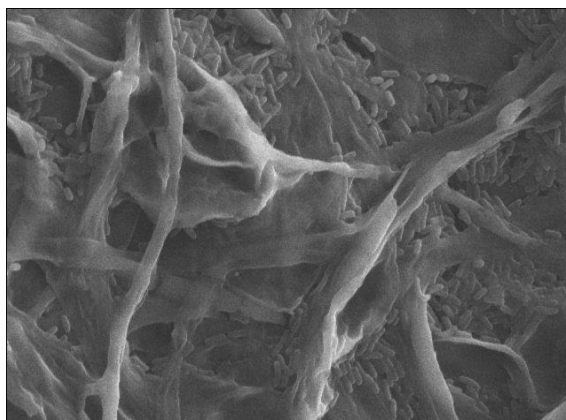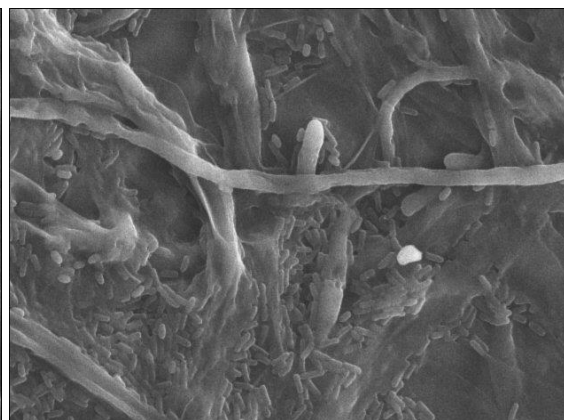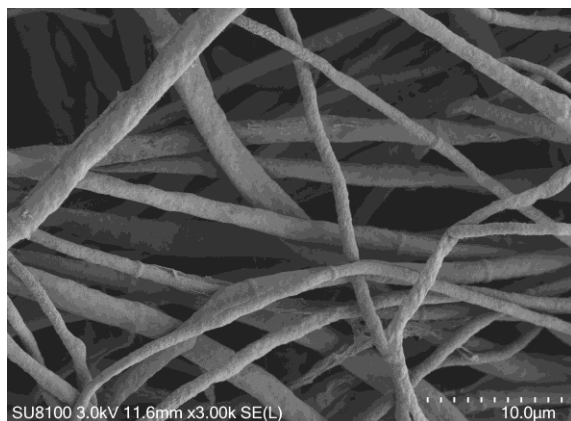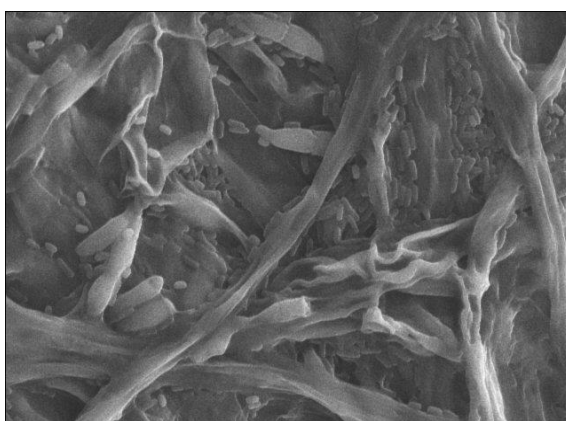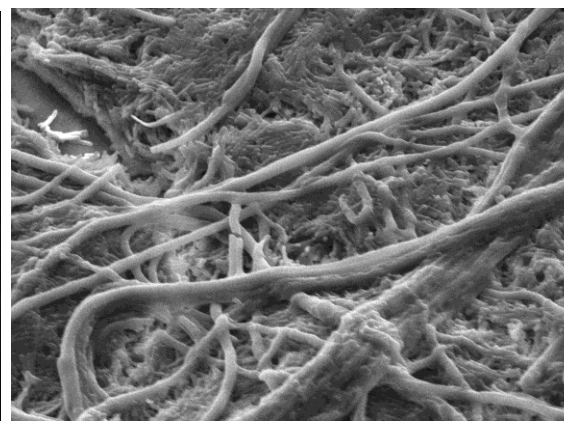

**Figure 5 (A-F)**

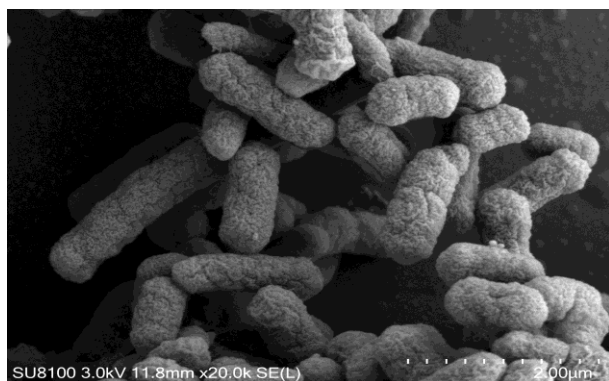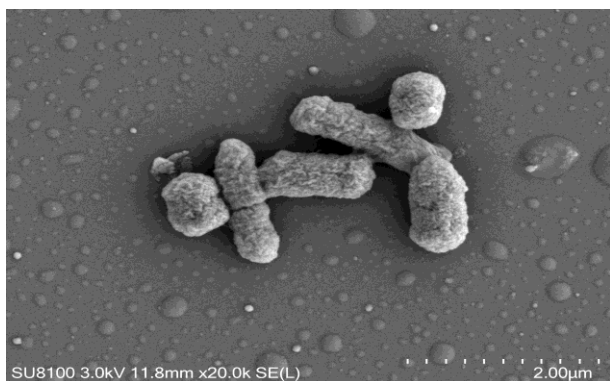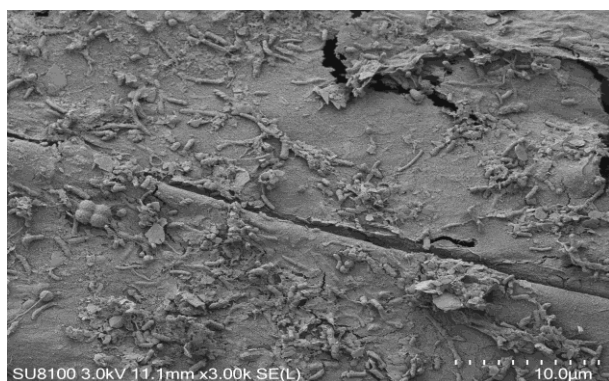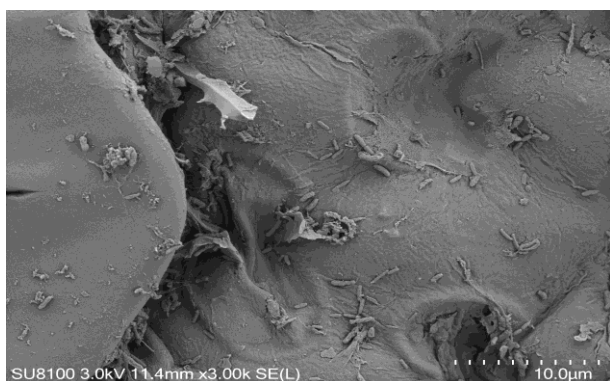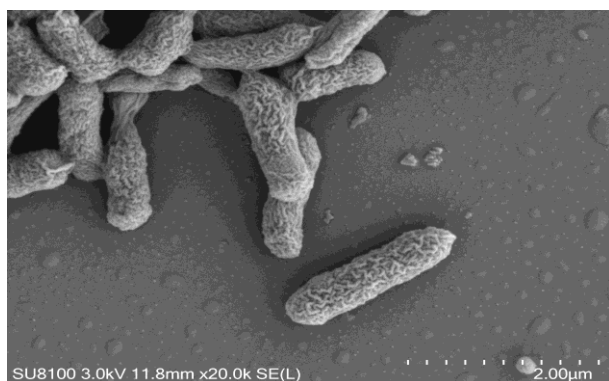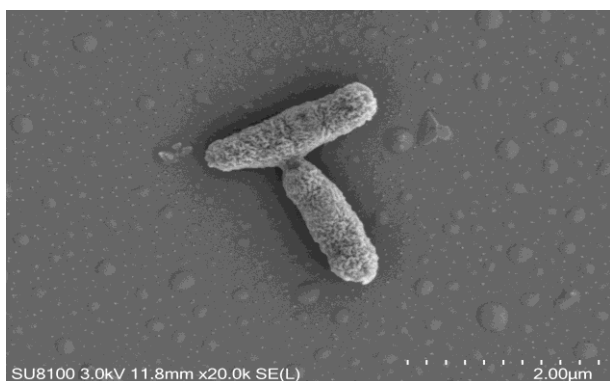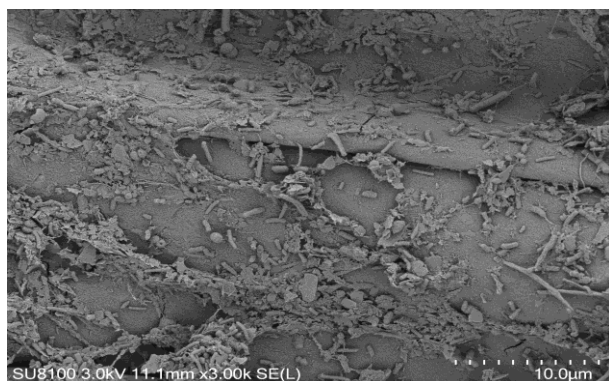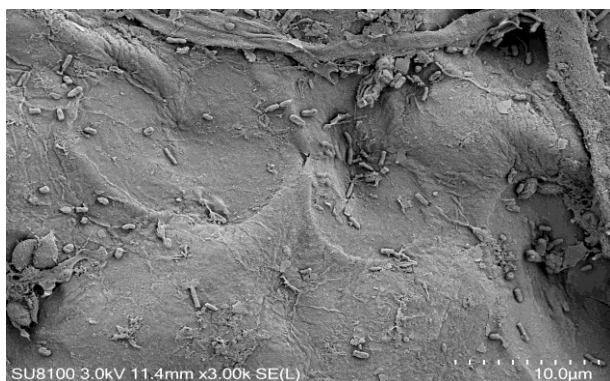

**Figure 7 (A-H)**

Supplement: Supplementary file 2 [file Presentation_1.PDF]
